# From theory to practice of designing for diversity: Applying intersectionality to improve HIV testing uptake

**DOI:** 10.1371/journal.pone.0335311

**Published:** 2025-10-27

**Authors:** Elham Ghasemi, Fatemeh Rajabi, Reza Majdzadeh

**Affiliations:** 1 Community Based Participatory Research Center, Tehran University of Medical Sciences, Tehran, Iran; 2 Center for Academic and Health Policy, Tehran University of Medical Sciences, Tehran, Iran; 3 School of Health and Social Care, University of Essex, Colchester, United Kingdom; University of Alabama at Birmingham, UNITED STATES OF AMERICA

## Abstract

**Introduction:**

Given the critical perspective of intersectionality and its potential to identify the causes of inequalities, it has been employed increasingly in studies related to health. Despite the rich theoretical evidence about intersectionality, there is a need to consider this approach empirically. This study aimed to apply the intersectionality in practice for health policy makers and researchers seeking to reduce health inequalities. In this regard, we described the development of an intersectionality-based and context-specific intervention focusing on HIV testing uptake among Afghan immigrants in Iran.

**Methods:**

This is an intervention development study. The intersectionality was used to design a peer-led intervention guided by the 2008 MRC framework. We undertook the following activities related to the three stages of the MRC framework: 1. Identifying the existing evidence (conducting a scoping review to investigate the application of intersectionality in designing and implementing health interventions; designing the checklist of applying the intersectionality in health interventions and programs); 2. Identifying and developing a program theory (conducting a realist review to identify why, how, and under what conditions peer interventions can improve HIV testing uptake among immigrants); and 3. Modeling process and outcomes (adapting the contextual factors identified by conducting a qualitative study and the realist review; extracting considerations regarding intersectionality principles using the checklist of applying the intersectionality in health interventions and programs; determining context specific, intersectionality-based and evidence-based intervention components for each of the intervention pathways).

**Results:**

According to considerations regarding checklist of application of intersectionality principles, the intervention at the different individual, organizational, and policy levels with multiple strategies should be designed to respond to needs/conditions affecting HIV testing uptake among immigrants. We determined the peer-led intervention features to improve the use of HIV testing services in Afghan immigrants following the intersectionality principles, target group needs, and contextual conditions aiming to modify power structures. Intervention strategies included HIV information provision, support, community-based services, and structural interventions.

**Conclusion:**

This study provides a practical framework for health planners and researchers seeking to reduce inequalities by presenting how intersectionality can influence the design of a health intervention. Accordingly, it is necessary to revisit the social relationships and power structures, determine the intervention components based on evidence tailored to the target group’s needs, and apply changes at different levels.

## Introduction

Despite all the efforts to reduce health inequalities, societies and governments still recognize it as a common concern. Given the complexity of health inequalities, it is crucial to develop comprehensive frameworks to address the social determinants of health involved in shaping health inequalities [1]. As a relatively new framework, intersectionality has been employed to understand and address the inequalities by identifying the intersections between multiple identities within social systems of power [2]. Although the term “intersectionality” was coined by Kimberlé Williams Crenshaw, this concept has stemmed from a long history of black feminism [3,4]. Several activists have discussed similar ideas about the interaction of different forms of inequality and interlocking oppressions of identity categories, such as Patricia Hill Colins [5], Lisa Bowleg [6], and Combahee River Collective-a group of Black lesbian socialist feminists formed in 1974 [7]. Intersectionality asserts that individual identities (gender, race/ethnicity, sex, …) overlap and have mutually interlocked effects that reflect power structures and macro-level forms of oppression and privilege [8]. Due to the planning and behavioral interventions at the individual level that have failed to address fundamental social, political, and economic determinants, current policies could fail to tackle health inequalities. Meanwhile, intersectionality is concerned with identifying and challenging power structures. Unlike intersectionality, whose primary focus is on the intersection and the simultaneous impacts of social identities within the structures of power, most of the existing policies consider the determinants separately; they are also mainly focused on the socio-economic characteristics that prevent the identification of other dimensions of identity leading to inequalities [3,9,10].

Numerous studies have reviewed the application of intersectionality in the field of health. Given the evidence, intersectionality has been used more frequently because of its evolutionary and critical perspective toward identifying the causes of inequalities and improving justice accordingly [11–13]. Although there is a rich theoretical background in the basics of intersectionality, it is still necessary to be tested in the health domain [14]. Findings from our previous scoping review revealed that intersectionality had been employed inadequately pertaining to the design and implementation of health interventions [15]. The lack of familiarity of researchers and policymakers with this approach and its methodological challenges has led to its limited application [10,15]. Intersectionality-based Policy Analysis Framework is considered a substantial effort to operationalize intersectionality. Hankivsky et al. introduced the nature, application, and principles of intersectionality, which include *“intersecting categories”, “multilevel analysis”, “power”, “reflexivity”, “time and space”, “diverse knowledge”, and “social justice & equity”* [3]. Although the development of this framework represents an essential step forward, it is still imperative to pursue further developments in order to emphasize methodological issues and their application in empirical studies [16].

Given the application of intersectionality and compliance with its principles, it is crucial to find out how intersectionality can facilitate the design and implementation of health programs. One of the global health issues that have been emphasized to end inequalities is HIV/AIDS [17]. The universal test-and-treat (UTT) strategy has been considered the cornerstone of the global AIDS control effort [18]. In spite of the substantial progress in this area, the progress to end HIV transmission is off track to reach the goal of ending AIDS “as a public health threat” by 2030. It is noteworthy that over half of new HIV infections currently occur among key populations and their sexual partners globally. Besides, some groups are dealing with more AIDS-related deaths and complications as well as less access to necessary interventions. However, most current HIV programs are not designed to meet the needs and realities of people living with these intersecting inequalities [17].

Immigrants are considered to be highly vulnerable to HIV/AIDS and are introduced as a key population in the 2025 AIDS targets [19]. According to the evidence, they are more likely to experience delays in HIV diagnosis and treatment compared to the host countries’ population [20–24]. Moreover, different researchers have investigated the effect of various individual, social, economic, and structural factors on immigrants’ access to HIV services [21,25,26]. Hence, international organizations have asked for efforts to reduce inequalities and help immigrant populations benefit from HIV services [27]. Various studies have highlighted Afghan immigrants’ poor health, economic, social, cultural, and political conditions as well as their limited access to health services [28–31]. However, there are a few studies on HIV and access to healthcare services among this population [28,29]. In a study conducted in Tehran (the capital of Iran), HIV prevalence using a rapid test was reported to be 0.2% (95% CI 1.2–0.005) with one HIV-positive case. Considering the limitations in the sampling methods and the small sample size, the researchers found it necessary to conduct future surveys to collect more detailed behavioral data to measure the real potential for the spread of HIV [32]. Considering the inequalities in the access of Afghan immigrants to health services in Iran [28,29,31], we aimed to apply the intersectionality approach to design the peer intervention to promote the use of HIV testing among immigrants.

## Materials and methods

This is an intervention development study. We used the intersectionality approach to design a peer-led intervention guided by the 2008 Medical Research Council (MRC) framework. MRC is an influential guide to researchers for developing and evaluating interventions with different interacting components [33]. The activities performed in the stages of the 2008 MRC framework are shown in Table 1 and more details are presented below.

**Table 1:** Activities to design the intersectionality-based peer-led intervention guided by MRC.

Stages	Activities
1) Identifying existing evidence	Conducting a scoping review to investigate the application of intersectionality in designing and implementing health interventions Designing the checklist of applying the intersectionality in health interventions and programs
2) Identifying and developing theory	Conducting a realist review to develop a program theory for identifying why, how, and under what conditions peer interventions can improve HIV testing uptake among this population.
3) Modeling process and outcomes	Conducting a qualitative study to identify contextual factors and solutions regarding Afghan immigrants’ access to HIV services in Iran. Adapting the contextual factors identified in the context of Iran and the realist review Determining the contextual factors that should be considered in the implementation of each intervention strategy Extracting considerations regarding intersectionality principles using the checklist of applying the intersectionality in health interventions and programs Determining context specific, intersectionality-based and evidence-based intervention components for each of the intervention pathways.

### Identifying existing evidence

In order to apply the intersectionality approach to design the peer intervention, it was first necessary to respond to the question: “What is the extent of the application of intersectionality in designing and implementing health interventions and programs?”. It could help us identify the characteristics of interventions that have been designed using intersectionality. Hence, we conducted a scoping review based on Arksey and O’Malley’s methodological framework [34] that included the following steps: 1: Identifying the research question; 2) Identifying relevant studies; 3) Selecting the studies; 4) Charting data; 5) Summarizing and reporting the results. In short, the articles published in databases of PubMed, Web of Science, Proquest, Embase, Scopus, Cochrane, and PsychInfo were screened based on their title and abstract and those that met the screening criteria, were reviewed in full. The inclusion criteria included: (1) to be a primary research, (2) conduct a health intervention or program, (3) clearly state the application of “*intersectionality”* in designing and implementing the intervention or program, (4) focus on health outcomes (5) address least two social identity variables (age, gender, ethnicity/race, social class, migration status, …) which could potentially be intersectional. Data about the application of principles of intersectionality was extracted and summarized. Details about the methodology of our scoping review have been reported previously [15]. We used the results of the scoping review regarding the application of intersectionality in health interventions and the characteristics of these interventions in the design phase of our peer intervention in a way that is based on the principles of intersectionality. In addition, a checklist was designed to evaluate the application of intersectionality in health interventions and programs. This checklist was prepared in order to facilitate the analysis of the existing health interventions and programs, as well as to help design, implement, and evaluate new interventions using the intersectionality approach by health researchers, planners, and policymakers. After reviewing the literature on intersectionality in the field of social sciences and health, the items related to the principles of intersectionality were extracted according to different stages of health planning, namely identification of the problem, design, implementation, and evaluation. Various versions of the checklist were reviewed during several expert panels, and the required amendments were applied accordingly. Finally, a 38-item checklist was developed based on the key principles of intersectionality, i.e., “intersecting categories”, “multilevel analysis”, “power”, “reflexivity”, “time & space”, “diverse knowledge”, and “social justice & equity”. In the present study, this checklist was used to moderate the considerations of observing the principles of intersectionality, which will be elaborated on in the modeling process and outcomes step.

### Identifying and developing theory

According to the “diverse knowledge” principle, intersectionality emphasizes the production of knowledge from different sources, such as qualitative or quantitative research, empirical or interpretive data, and indigenous knowledge [35]. Using realist review, as a theory-based approach, helps us to synthesize several types of existing evidence [36]. By developing Context-Mechanism-Outcome Configurations (CMOCs) during the process of analysis and generating program theory, this type of review can identify how an intervention or action under certain contextual circumstances (C) may trigger a mechanism (M) to achieve a given outcome (O) [36,37]. We conducted a realist review to develop a program theory for identifying why, how, and under what conditions peer interventions can improve HIV testing uptake among immigrants. First, an initial program theory was developed regarding the mechanism of peer interventions and contextual factors. Then, this theory was tested through a review of evidence. The following inclusion criteria were considered for this realist review. 1. different types of primary studies, 2. interventional or non-interventional studies with different designs investigating the international immigrant population, and 3. the effect of peers (as the provider of education, support, or counseling) on HIV testing uptake. The search resources included the following electronic databases: PubMed, Web of Science, Scopus, Embase, and Cochrane; the websites of related international organizations: The Joint United Nations Programs on HIV/AIDS (UNAIDS), the World Bank, Global Fund, WHO, and International Organization for Migration (IOM); as well as the reference list of the related studies. After employing the Preferred Reporting Items for Systematic Reviews and Meta-Analyses (PRISMA) process to select the relevant studies, the researchers conducted data extraction to explain the concepts of initial program theory and find potential relationships between them. In addition, the quality of the selected studies was evaluated. Two independent reviewers were assigned to conduct the steps of selecting studies, extracting data, and evaluating the quality of the evidence. They would also seek agreement in case of any disagreements. The content analysis method was used to analyze the data. Then, strategies, intervention mechanisms, and influential contextual factors were identified during the data categorization. Furthermore, data coding and categorization procedures were discussed, and the research team reached a consensus during several meetings. Eventually, the relationship between these concepts and different pathways was determined in theory and the refined program theory was presented in our previous article [38].

### Modelling process and outcomes

We used the causal model to link the contextual factors affecting the pathways of intervention strategies. Intervention features were mapped onto the causal pathway. This model was revised and finalized during the discussion of the research team in different meetings.

Context often pertains to anything external to the intervention that plays a critical role as a barrier or facilitator to its implementation, or its effects [39]. Given the emphasis of intersectionality on the importance of context in the development of experiences of oppression and privilege [8], we initially conducted a qualitative study between June 2018 and April 2020 to identify contextual factors and also solutions regarding Afghan immigrants’ access to HIV services in the context of Iran. The methodology details are presented in our previous paper [40]. Briefly, the study participants included three groups of stakeholders: Afghan immigrants diagnosed with or at risk of HIV/AIDS (n = 8), service providers (n = 8), and policymakers/managers/experts (n = 9). The participants were selected using purposive and snowball sampling. The data were then collected through semi-structured interviews with different stakeholders by the first author. In addition, a male researcher trained for qualitative interviews assisted in conducting interviews with male immigrants. Data analysis was performed based on the content analysis method. In the qualitative study, we aimed to respect the intersectionality principles: The principle of “diversity of knowledge” emphasizes the inclusion of perspectives of people who are typically marginalized or excluded in the production of knowledge [35]. In this regard, we considered the opinions of different groups of stakeholders, particularly the voices of immigrants, as a marginalized and disadvantaged group. Moreover, interviews were carried out across health systems and community levels in keeping with the “multiple-level analysis” principle of intersectionality. As a result, several factors were explored at different individual/interpersonal, organizational, and structural levels. More details were reported in our previous article [40]. Furthermore, the lens of intersectionality was used to identify intersectional stigma and discrimination in accessing HIV services among Afghan immigrants. We found that the intersection of different layers of stigma should be taken into account while designing and implementing HIV prevention and treatment programmes. This intersectional qualitative study is presented in our previous paper [41].

In order to adapt the context of intervention and contextual factors of the causal model provided by the realist review, the contextual factors explored in the qualitative study (individual/interpersonal, organizational, and structural factors), as well as the contextual factors identified in the realist review, were classified and compared to examine the potential consistency of these contextual factors.

In the next step, after determining the contextual factors that should be considered in implementing each intervention strategy, the intervention components were determined for each of the intervention pathways. At this stage, the results of the scoping review regarding the characteristics of interventions based on intersectionality, as well as the items of the checklist of application of intersectionality, were addressed so that the intervention was designed based on the principles of intersectionality. In this regard, we extracted considerations related to intersectionality principles while designing and implementing peer intervention. Finally, context-specific, intersectionality-based and, evidence-based intervention components were determined to improve HIV testing uptake among Afghan immigrants in Iran.

### Ethical considerations

This study was reviewed and approved by the Ethics Committee of Tehran University of Medical Sciences, Iran (IR.TUMS.VCR.REC.1396.4171). We confirm that all methods were performed in accordance with the relevant guidelines and regulations and that all of the processes were approved by the institutional review board. The oral and written consent was obtained from all participants in the qualitative phase of the study. They were assured of confidentiality and anonymity about the information that they provided. In this regard, we conducted individual interviews in a private room. Moreover, no personally identifying information was collected. In the analysis and publication of results, the findings were completely anonymous and analyzed and published based on the group data.

## Results

### Identifying existing evidence

We previously reported the details about the results of our scoping review [15]. 2677 articles were found through reviewing the target databases. After removing the duplicated ones and screening the titles and abstracts of 1601 studies, 107 articles were selected to be reviewed in detail and, four articles could meet the criteria. We focus on findings that were used to design the peer intervention: 1) Intersectionality focuses on vulnerable populations. So, the marginalized people were considered as the target group of interventions. 2) The studies attempted to consider the intersection of these co-occurring social identities and conditions through the intersectionality framework to achieve a more comprehensive understanding of the issue and identify the target groups accurately. 3) Including the viewpoints of marginalized individuals and groups who are excluded from knowledge production can help to reduce the pressure of power. In this regard, the application of participatory approaches can be useful. 4) Most of the included studies have conducted the interventions at the micro level (personal and interpersonal relationship), and have not aimed at bringing about any changes at the structural levels. Therefore, more attention to power structures at meso and macro levels to reach the power balance and establish social justice is necessary [15].

### Identifying and developing theory

By developing the context-mechanism-outcome configurations during the process of analysis and generating a program theory, our realist review identified the peer interventions worked through four pathways: Following the improvement of communications (as a proximal mechanism): 1) increasing awareness, 2) reduced stigma, 3) improved support, and 4) increased access to services could lead to improved HIV testing uptake among immigrants. Peer interventions with multiple strategies (providing HIV information, providing support, community-based strategy, and providing HIV testing services) to be designed and implemented considering the barriers to HIV testing and also moving beyond one-size-fits-all approaches can successfully improve the immigrants’ HIV testing uptake. The HIV information strategy focuses on implementing different educational methods and adapting educational content to immigrants’ cultural conditions, educational needs, and existing barriers. Moreover, the “providing support” strategy highlights peers’ role as supporters or advocators of immigrants through providing informational support and advice, contacting social network members, accompanying immigrants to HIV testing centers, and defending their peers while experiencing potential violence. The community-based strategy encompasses different approaches, such as using CBPR approaches and chain referral methods in studies, as well as providing outreach services. Regarding the providing HIV testing services strategy, peers usually play the role of someone who suggests referring to the testing center and performing HIV tests. In addition, contextual factors were categorized into three groups: individual/interpersonal factors (language, values, and beliefs, socio-economic status, immigration status, fear and stigma, gender), health service-related factors (accessibility, availability, acceptability, affordability), and structural factors (regulations and policies, supportive resources, discrimination) [38].

### Modeling process and outcomes

**Fig 1:**
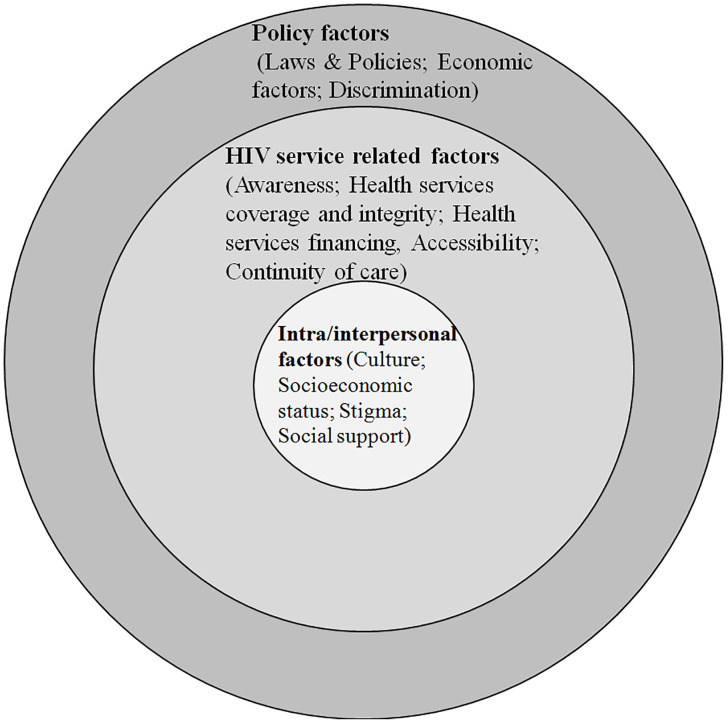
The intersection of factors influencing Afghan immigrants’ access to HIV testing services.

At this stage, the qualitative study resulted in realist review, and scoping review to map the causal model and determine the intervention features. The qualitative study led to contextual factors affecting the improvement of HIV testing uptake among Afghan immigrants in Iran. The findings of this study have been published in our previous article [40]. In total, 25 individuals participated in this study. The average age of immigrants was 40 years (range: 23–78). Three of the participating immigrants were female, and the rest were male. The immigrants had been living in Iran for 30.5 years on average within the range of 23–36 years. Moreover, most of them were married and undocumented, and the majority held low education degrees. They also reported poor working conditions, and most of them did not have any access to health insurance. The average age of service providers and policymakers was 38.37 years (range: 28–51) and 51.55 years (range: 39–74), respectively. Moreover, they held educational degrees in different disciplines. The factors influencing Afghan immigrants’ access to HIV/AIDS services were categorized into 3 themes that were extracted from 9 categories: 1. Cultural (cultural similarities and differences, values and beliefs); 2. Psychosocial (social support, stigma, and discrimination); and 3. Service delivery-related factors (awareness, health services coverage and integrity, health services financing, accessibility, and continuity of care). According to the qualitative analysis via intersectionality lens, immigrants who had language barriers in communication were in poor economic, social, cultural, and supportive conditions, and had experienced stigma and discrimination; hence, they were more likely to confront problems with HIV testing services uptake. Such experiences were formed in the context of barriers at the level of service delivery, as well as the impact of immigration structures and regulations, educational policies, and poor intersectoral collaborations. In other words, in the context where the HIV and migration-related stigma and discrimination have been experienced at different levels (macro: policymaking; meso: social and health services; and micro: intra and interpersonal), immigrants who had communication, support, and economic barriers, experienced more deprivation of access to HIV services. This issue showed the interlocked effects of power structures and social relations at different levels (Fig 1). Furthermore, the participants proposed some solutions to improve immigrants’ access to such services. These suggestions include raising HIV awareness (particularly regarding the nature of the disease, prevention, and diagnosis), providing financial and non-financial support, reducing stigma and discrimination at multiple levels, as well as providing outreach and peer-support services according to Afghan immigrants’ needs and cultural values.

In the next step, the results of comparing the contextual factors affecting the use of HIV testing in the realist review of the evidence with the factors identified in our qualitative study indicated the similarities of the factors (Table 2).

**Table 2:** Contextual factors influencing HIV testing uptake extracted from evidence and context of Iran.

Contextual factors	Realist review evidence	Qualitative study findings
**Intra/interpersonal factors**	Language and cultural barriers; Poor knowledge about HIV and available services; Poor economic and occupational conditions; Illegal immigration and residential mobility; Fear and stigma	Cultural factors (language, values, …); poor knowledge about HIV and available services; Low literacy levels; Poor economic and occupational conditions; Residential mobility; Fear and stigma; Poor social support
**Health service related factors**	Geographical accessibility issues; Outreach services as the facilitator; Limited working hours for HIV testing and long waiting time; Providing services based on immigrants’ needs; Cultural and language conditions; Peers’ characteristics as the facilitator of service acceptability; Affordability of services; poor economic conditions, lack of health insurance, and costly HIV testing	Service provision location (time and distance); Improper provision of integrated services; Lack of services based on immigrants’ needs and characteristics; Mistrust; Lack of continuity of services due to uncertain residence issues and transfer of service providers; Costs; Occupational facilitators; Free HIV tests; Incentive for peers
**Structural factors**	Immigration regulations and fear of deportation Criminalization of most aspects of sex work Public health regulations of periodical HIV/STI testing for high-risk individuals as a facilitator Insurance policies and costly HIV testing for immigrants The facilitating role of media and NGOs, as well as the contribution of community-based organizations Discrimination	Immigration regulations and fear of deportation The facilitating role of providing free HIV testing to immigrants irrespective of their migration status Poor educational policies Improper intersectoral collaborations The facilitating role of community participation (peers and NGOs) Discrimination due to lack of antidiscrimination laws

We addressed the results of the realist review to design the intervention: the common features of successful interventions in improving HIV test uptake among immigrants included: 1) They used multiple strategies; 2) They were designed and implemented based on the immigrants’ needs and conditions, and; 3) They have addressed the barriers of access to HIV testing services. Hence, we considered these features while designing the peer interventions. In addition, researchers identified multiple intervention strategies (providing HIV information, providing support, community-based strategy, and providing HIV testing services) and mechanisms (increasing awareness, reducing stigma, improving support, and increasing access to services) to use in mapping the causal model [38].

In the next step, the research team reviewed the findings of the scoping review regarding the application of intersectionality in health interventions and the characteristics of these interventions (mentioned in the results section of the scoping review) as well as the checklist items of the use of intersectionality in health interventions and programs, and then extracted considerations for intersectionality principles while determining peer intervention features. These considerations are shown in Box 1.

**Box 1. Considerations regarding checklist of application of intersectionality principles while designing and implementing peer intervention**

- The intervention/program should be designed and implemented based on the intersection of immigrants’ identities and economic, social, and political conditions of the context. In other words, the interventions with multiple strategies should be designed to respond to intersectional needs/conditions affecting HIV testing uptake among immigrants.
- Interventions should be implemented at the different individual, organizational, and policy levels.
- According to the intersectionality principles, different groups of stakeholders are required to design and implement the intervention/program, including immigrants, service providers, and policymakers.
- It is necessary for HIV service providers and policymakers to recognize Afghan immigrants as a group with the most vulnerability and also to be informed about the cultural characteristics of immigrants.
- According to the intersectional stigma towards Afghan immigrants, necessary conditions should be considered in the design and implementation of the intervention to maintain the confidentiality of the immigrants’ information and required training should be provided to peers in this regard.
- The educational content, communication channels, and implementation methods (individual/group) should be conducted and tailored to immigrants’ conditions to increase knowledge and provide support to immigrants (flexible educational and support methods should be observed).

**Fig 2:**
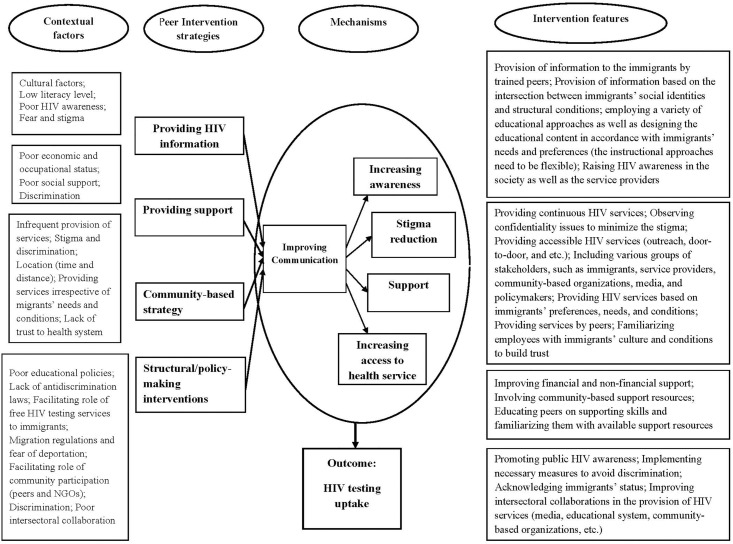
Causal model of peer intervention to improve HIV testing uptake among Afghan immigrants in Iran.

Lastly, the research team reviewed the intervention features for each intervention pathway and finalized them in a way that was context-specific and based on the considerations of intersectionality principles. These features are presented in Fig 2.

## Discussion

The present study provides an example of the application of intersectionality through designing a peer intervention to improve the use of HIV testing among Afghan immigrants in Iran. Using the 2008 MRC framework, we could develop a theory-based and evidence-based intervention and determine a context-based causal pathway to suggest peer intervention features. The researchers intended to assess the context by reviewing the evidence as well as exploring stakeholders’ perspectives and addressing the factors at different levels. As an example of the application of intersectionality in the field of health, this study can be helpful in enriching our knowledge regarding the application of intersectionality in practice.

Using the 2008 MRC framework helped us apply intersectionality in a comprehensive, flexible, and non-linear approach [42]. These MRC framework features are consistent with the application of intersectionality principles that follow an iterative and flexible approach. In this study, researchers addressed the principles of intersectionality to design the peer intervention were addressed in several ways, which we discuss below:

Oppressed and marginalized people are regarded as one of the significant tenets of intersectionality concerning public health [9]. In this regard, the present study has focused on Afghan immigrants as a vulnerable and marginalized group in Iran. Afghan immigrants, as the largest group of immigrants in Iran, typically suffer from poor economic, social, and occupational conditions; besides, previous systematic reviews have indicated their deprivation of social and health services [28,29]. The findings of our qualitative study revealed that the majority of these immigrants reported low literacy levels as well as inappropriate economic and employment status. Most of them did not have a valid migration card and health insurance; furthermore, they faced different obstacles in accessing HIV services [40].

Challenging the existing power structures and valuing the voices of vulnerable groups are emphasized in the intersectionality approach [3]. Accordingly, the present study aimed to listen to the immigrants’ voices, balance the power, and pay attention to the power structures through the following ways:

Firstly, according to the results of the realist review, researchers identified a significant number of barriers to improving immigrants’ HIV testing uptake. Adapting the contextual factors identified in our qualitative study during the modeling phase enabled us to design a context-based intervention focusing on the needs of Afghan immigrants and the structural factors of HIV service delivery in Iran’s health system. Meanwhile, the intersectionality principles of “diverse knowledge”, “multilevel analysis”, and “power” were addressed. Besides, identifying the immigrants’ lived experiences about accessing HIV services could particularly contribute to paying attention to their voices. The relationship between power and knowledge production is highlighted within the intersectionality approach. In order to reduce the power pressure in health planning or policies, it is crucial to consider the perspectives and worldviews of marginalized groups who are excluded from the knowledge production process [35]. Furthermore, researchers employed the realist review to examine the evidence to identify the strategies of successful peer interventions, in line with the principle of “diverse knowledge”. It was also intended to pay attention to the contextual factors affecting the efficiency of the interventions in the existing evidence. These strategies were then used in designing the intervention after approving the adaptation of this evidence to the context of Iran.

Secondly, the “*peer*” interventions were selected in accordance with the principles of intersectionality. Hence, in the context where there are discriminatory structures, especially towards HIV and Afghan immigrants, the findings of the realist review highlighted that the interventions through the participation of peers, who were among the target group with some similarities in terms of social identities, could lead to a power balance that might play a critical role in the success of the interventions. Peers’ ability to communicate with immigrants on an equal level by understanding their challenges can help the success of these peer interventions. Equal relationships put peers in a position where they can play the role of a mediator between the client and the service provider, depending on the needs and the existing situation so as to develop the balance in power [43]. On the other hand, one of the performance characteristics of peers is encouraging the target populations in participatory learning processes, and empowering them. Since empowerment is crucial in reducing health inequalities and accessing their health care services, such interventions target marginalized and socially disadvantaged groups [44]. Moreover, the supportive peer relationship can provide a more critical understanding of how personal experience is related to social and political struggles and actively promote “social justice” [44]. It seems that it is necessary to pay more attention to the value of the peers’ role. Moreover, it is suggested to define systematically their position, especially in the context of health systems with limited resources.

Thirdly, we proposed intervention strategies at individual levels (raising awareness, providing support, and involving the target group in providing testing services) as well as structural interventions at policy levels. Therefore, it was recommended to promote HIV-related educational policies, implement discrimination prevention guidelines, acknowledge immigrants at the policy level, and promote intersectoral collaboration while providing HIV services. One might hope to reduce inequalities in practice by proposing changes at the policy levels; similarly, disregarding this issue and designing health interventions at individual levels has been mentioned as one of the obstacles to the successful implementation of existing policies in reducing inequalities. On the other hand, the intersectionality perspective has a significant potential to reduce inequalities by emphasizing the identification and challenging power structures [10]. In intersectionality-based studies, interventions should focus on systems and power structures, including health care systems, public policy, regulations, education, and the economy, rather than highlighting individuals and groups [45]. In this regard, it has been confirmed that in advocacy-based peer interventions, behavior changes at the individual level or health outcomes are possible only if changes occur at the levels of social power structures [44]. The findings of our scoping review also indicated that most studies disregard the impacts of power structures, such as policies in emphasizing or responding to the problem [15].

Given the limited application of intersectionality in health interventions, it is essential to employ the instruments and processes to apply this approach step by step, from problem identification to designing and implementing the intervention. It can further help familiarize researchers and policymakers with this issue and operationalize this approach in designing and implementing health programs. Meanwhile, it is recommended to observe the intersectionality principles. The operationalization of intersectionality would be facilitated by the participation of diverse groups of stakeholders (especially the target group), highlighting the intersection of conditions at different micro, meso, and macro levels, the identification of power structures, and arranging evidence-based strategies for interventions in accordance with the context conditions as well as the intersection of target population’ identities.

### Strengths and limitations of the study

Provided that the application of intersectionality is associated with various challenges, this study intended to illustrate the operational use of intersectionality by reporting a case of designing peer interventions aimed to improve the use of HIV testing in a group of immigrants; it also seems that health planners can employ this approach to design interventions through intersectionality approach. According to the evidence, it is necessary to conduct further research on enhancing designs and methods that can effectively include the principles of intersectionality [46]. In this regard, a checklist was employed in the present study to evaluate the application of intersectionality in different stages of health planning. This checklist can be implemented as an objective instrument to observe the principles of intersectionality in designing, implementing, and evaluating interventions. In addition to designing the intervention at the individual and service delivery levels, we proposed to modify the power structures at the macro level that have received less attention in previous studies that applied intersectionality [15]. Moreover, guiding the development of the intervention based on the MRC framework made us finally suggest an evidence-based intervention. However, the application of intersectionality principles required spending time and resources. Due to these limitations, we could only conduct the study until determining the intervention features for each intervention pathway and finalized them in a way that was context-specific and based on the considerations of intersectionality principles. According to the MRC framework, it is necessary to evaluate the developed intervention by conducting an interventional study [33]. On the other hand, various evidences have been cited that applying the MRC framework is time-consuming and costly in terms of research resources [42,47,48]. Therefore, researchers and policymakers who intend to use intersectionality in the design, implementation, and evaluation of interventions and health programs should consider allocating financial resources and considering enough time to apply intersectionality.

## Conclusions

Intersectionality has been increasingly used by researchers as an emerging approach in the field of health. However, the practical application of intersectionality is still regarded as a research gap and requires further research in this field. The present study highlighted the applicability of intersectionality in designing a health intervention guided by the MRC framework as a practical and flexible approach. Accordingly, it is necessary to pay attention to developing the intervention considering the relationships and structures of the context, determine the intervention components based on the evidence and needs of the target group, as well as apply modifications at different levels.
