# Supplementary material for: From theory to practice of designing for diversity: Applying intersectionality to improve HIV testing uptake
Source: PLoS One. 2025 Oct 27;20(10):e0335311. doi: 10.1371/journal.pone.0335311 (PMC12558453; doi:10.1371/journal.pone.0335311)

PLOS ONE

From theory to practice of designing for diversity: applying intersectionality to improve HIV testing uptake

--Manuscript Draft--

**Manuscript Number:**	PONE-D-23-10867
**Article Type:**	Research Article
**Full Title:**	From theory to practice of designing for diversity: applying intersectionality to improve HIV testing uptake
**Short Title:**	applying intersectionality to improve HIV testing uptake
**Corresponding Author:**	Reza Majdzadeh<br>University of Essex<br>Colchester, UNITED KINGDOM
**Keywords:**	HIV; AIDS; Intersectionality; health inequalities; health interventions; immigrants
**Abstract:**	Introduction: Given the critical perspective of intersectionality and its potential to identify the causes of inequalities, it has been employed increasingly in studies related to health. Despite the rich theoretical evidence about the intersectionality, there is a need to consider this approach empirically. Consequently, researchers aimed to operationalize intersectionality and conducted a multi-stage study proposing interventions to seek improvement in taking HIV test among Afghan immigrants in Iran.<br>Methods: The study was designed in five stages: 1- conducting a qualitative study to identify potential barriers and provide solutions regarding Afghan immigrants' access to HIV services in the context of Iran, 2- conducting a realist review to identify the strategies and mechanisms of successful interventions in the evidence and contextual factors affecting HIV testing uptake among international immigrants, 3- adapting the contextual factors that have been identified in the evidence to the context of Iran, 4- conducting the scoping review of interventions based on the intersectionality approach and designing a checklist for the application of intersectionality in designing and implementing health interventions, and 5- designing intervention to improve Afghan immigrants' access to HIV testing services.<br>Results: According to our findings, the peer-led intervention was designed to improve the use of HIV testing services in Afghan immigrants. Intervention strategies included HIV information provision, support, community-based services, and structural interventions. The intervention was developed following the intersectionality principles, target group needs, and contextual conditions aiming to modify power structures.<br>Conclusion: This study provides a practical framework for health planners and researchers who are seeking to reduce inequalities by presenting how intersectionality can be used in designing a health intervention. Accordingly, it is necessary to revisit the social relationships and power structures, determine the intervention components based on evidence tailored to the target group needs, and apply changes at different levels.
**Order of Authors:**	Elham Ghasemi
	Fatemeh Rajabi
	Reza Majdzadeh
**Additional Information:**	
**Question**	**Response**
**Financial Disclosure**<br><br>Enter a financial disclosure statement that describes the sources of funding for the work included in this submission. Review the submission guidelines for detailed requirements. View published research	This work was supported by Tehran University of Medical Sciences (TUMS), Grant number [96-03-62-36567]. The funder had no role in study design, data collection and analysis, decision to publish, or preparation of the manuscript.

Powered by Editorial Manager® and ProduXion Manager® from Aries Systems Corporation

articles from *PLOS ONE* for specific examples.<br><br>This statement is required for submission and **will appear in the published article** if the submission is accepted. Please make sure it is accurate.<br><br>**Unfunded studies**<br>Enter: *The author(s) received no specific funding for this work.*<br><br>**Funded studies**<br>Enter a statement with the following details:<br>• Initials of the authors who received each award<br>• Grant numbers awarded to each author<br>• The full name of each funder<br>• URL of each funder website<br>• Did the sponsors or funders play any role in the study design, data collection and analysis, decision to publish, or preparation of the manuscript?<br>• **NO** - Include this sentence at the end of your statement: *The funders had no role in study design, data collection and analysis, decision to publish, or preparation of the manuscript.*<br>• **YES** - Specify the role(s) played.<br><br>* typeset	
**Competing Interests**<br><br>Use the instructions below to enter a competing interest statement for this submission. On behalf of all authors, disclose any competing interests that could be perceived to bias this work—acknowledging all financial support and any other relevant financial or non-financial competing interests.<br><br>This statement is required for submission and **will appear in the published article** if the submission is accepted. Please make sure it is accurate and that any funding sources listed in your Funding Information later in the submission form are also declared in your Financial Disclosure statement.	The authors have declared that no competing interests exist.

Powered by Editorial Manager® and ProduXion Manager® from Aries Systems Corporation

View published research articles from *PLOS ONE* for specific examples.<br><br>**NO authors have competing interests**<br><br>Enter: *The authors have declared that no competing interests exist.*<br><br>**Authors with competing interests**<br><br>Enter competing interest details beginning with this statement:<br><br>*I have read the journal's policy and the authors of this manuscript have the following competing interests: [insert competing interests here]*<br><br>* typeset	
**Ethics Statement**<br><br>Enter an ethics statement for this submission. This statement is required if the study involved:<br><br>• Human participants<br>• Human specimens or tissue<br>• Vertebrate animals or cephalopods<br>• Vertebrate embryos or tissues<br>• Field research<br><br>Write "N/A" if the submission does not require an ethics statement.<br><br>General guidance is provided below. Consult the submission guidelines for detailed instructions. **Make sure that all information entered here is included in the Methods section of the manuscript.**	This study was reviewed and approved by the Ethics Committee of Tehran University of Medical Sciences, Iran (IR.TUMS.VCR.REC.1396.4171). We confirm that all methods were performed in accordance with the relevant guidelines and regulations and all of the processes approved with the institutional review board. The written informed consent was obtained from all participants in the qualitative phase of the study. They were assured of confidentiality and anonymity about the information that they provided.

Powered by Editorial Manager® and ProduXion Manager® from Aries Systems Corporation

**Format for specific study types**<br><br>**Human Subject Research (involving human participants and/or tissue)**<br>• Give the name of the institutional review board or ethics committee that approved the study<br>• Include the approval number and/or a statement indicating approval of this research<br>• Indicate the form of consent obtained (written/oral) or the reason that consent was not obtained (e.g. the data were analyzed anonymously)<br><br>**Animal Research (involving vertebrate animals, embryos or tissues)**<br>• Provide the name of the Institutional Animal Care and Use Committee (IACUC) or other relevant ethics board that reviewed the study protocol, and indicate whether they approved this research or granted a formal waiver of ethical approval<br>• Include an approval number if one was obtained<br>• If the study involved *non-human primates*, add *additional details* about animal welfare and steps taken to ameliorate suffering<br>• If anesthesia, euthanasia, or any kind of animal sacrifice is part of the study, include briefly which substances and/or methods were applied<br><br>**Field Research**<br><br>Include the following details if this study involves the collection of plant, animal, or other materials from a natural setting:<br>• Field permit number<br>• Name of the institution or relevant body that granted permission	
**Data Availability**<br><br>Authors are required to make all data underlying the findings described fully available, without restriction, and from the time of publication. PLOS allows rare exceptions to address legal and ethical concerns. See the PLOS Data Policy and FAQ for detailed information.	Yes - all data are fully available without restriction

A Data Availability Statement describing where the data can be found is required at submission. Your answers to this question constitute the Data Availability Statement and **will be published in the article**, if accepted.<br><br>**Important:** Stating 'data available on request from the author' is not sufficient. If your data are only available upon request, select 'No' for the first question and explain your exceptional situation in the text box.<br><br>Do the authors confirm that all data underlying the findings described in their manuscript are fully available without restriction?	
**Describe where the data may be found in full sentences. If you are copying our sample text, replace any instances of XXX with the appropriate details.**<br><br>• If the data are **held or will be held in a public repository**, include URLs, accession numbers or DOIs. If this information will only be available after acceptance, indicate this by ticking the box below. For example: *All XXX files are available from the XXX database (accession number(s) XXX, XXX.)*.<br>• If the data are all contained **within the manuscript and/or Supporting Information files**, enter the following: *All relevant data are within the manuscript and its Supporting Information files.*<br>• If neither of these applies but you are able to provide **details of access elsewhere**, with or without limitations, please do so. For example:<br><br>*Data cannot be shared publicly because of [XXX]. Data are available from the XXX Institutional Data Access / Ethics Committee (contact via XXX) for researchers who meet the criteria for access to confidential data.*<br><br>*The data underlying the results presented in the study are available from (include the name of the third party*	All relevant data are within the paper.

and contact information or URL).<br>• This text is appropriate if the data are owned by a third party and authors do not have permission to share the data.<br><br>* typeset	
Additional data availability information:	

Powered by Editorial Manager® and ProduXion Manager® from Aries Systems Corporation

Manuscript Click here to access/download;Manuscript;Manuscript.docx

Title page

From theory to practice of designing for diversity: applying intersectionality to improve HIV testing uptake

Authors' names and affiliations:

1-**Elham Ghasemi**: Community-Based Participatory Research Center, Tehran University of Medical Sciences, Tehran, Iran. Email:elham.ghasemi1@gmail.com

2- **Fatemeh Rajabi**: 1) Community-Based Participatory Research Center, Tehran University of Medical Sciences, Tehran, Iran. 2) Center for Academic and Health Policy, Tehran University of Medical Sciences, Tehran, Iran. Email: frajabi@tums.ac.ir

3- **Reza Majdzadeh*(Corresponding author)**: Interdisciplinary Research and Practice Division, School of Health and Social Care, University of Essex, Colchester, UK.

Email: reza.majdzadeh@essex.ac.uk

ORCID: 0000-0001-8429-5261

Address: Kimmy Eldridge Building, University of Essex, Wivenhoe park, Colchester, CO4 3SQ, UK.

Tel: (+44) 01206 87 – 4466

Author's Contributions:

EG: Conceptualization, Methodology, Investigation, Formal analysis and Writing-Original draft, Review and Editing**. FR:** Conceptualization, Methodology, Formal analysis, Supervision, Project administration, and Writing- Original draft, Review and Editing**. RM:** Conceptualization, Methodology, Formal analysis, Supervision, Project administration, Funding acquisition and Writing- Original draft, Review and Editing**.**

Abstract

Introduction: Given the critical perspective of intersectionality and its potential to identify the causes of inequalities, it has been employed increasingly in studies related to health. Despite the rich theoretical evidence about the intersectionality, there is a need to consider this approach empirically. Consequently, researchers aimed to operationalize intersectionality and conducted a multi-stage study proposing interventions to seek improvement in taking HIV test among Afghan immigrants in Iran.

Methods: The study was designed in five stages: 1- conducting a qualitative study to identify potential barriers and provide solutions regarding Afghan immigrants' access to HIV services in the context of Iran, 2- conducting a realist review to identify the strategies and mechanisms of successful interventions in the evidence and contextual factors affecting HIV testing uptake among international immigrants, 3- adapting the contextual factors that have been identified in the evidence to the context of Iran, 4- conducting the scoping review of interventions based on the intersectionality approach and designing a checklist for the application of intersectionality in designing and implementing health interventions, and 5- designing intervention to improve Afghan immigrants' access to HIV testing services.

Results: According to our findings, the peer-led intervention was designed to improve the use of HIV testing services in Afghan immigrants. Intervention strategies included HIV information provision, support, community-based services, and structural interventions. The intervention was developed following the intersectionality principles, target group needs, and contextual conditions aiming to modify power structures.

Conclusion: This study provides a practical framework for health planners and researchers who are seeking to reduce inequalities by presenting how intersectionality can be used in designing a health intervention. Accordingly, it is necessary to revisit the social relationships and power structures, determine the intervention components based on evidence tailored to the target group needs, and apply changes at different levels.

Keywords: HIV; AIDS; Intersectionality; health inequalities; health interventions; immigrants

Introduction:

Despite all the efforts to reduce health inequalities, this issue is still regarded as a common concern by societies and governments. Given the complexity of health inequalities, it is crucial to development of comprehensive frameworks to address the social determinants of health involved in shaping health inequalities [1]. As a relatively new framework, intersectionality has been employed to understand and address the inequalities by identifying the intersections between multiple identities within social systems of power [2]. The term "intersectionality" was originally coined by Kimberlé Williams Crenshaw, and used to refer to the double discrimination of racism and sexism faced by Black women [3]. Intersectionality asserts that individual identities (gender, race/ethnicity, sex, …) overlap and have mutually interlocked effects that reflect power structures and macro-level forms of oppression and privilege [4]. Due to the planning and behavioral interventions at the individual level that have failed to address fundamentally social, political, and economic determinants, current policies could fail to tackle health inequalities. Meanwhile, intersectionality is concerned with identifying and challenging power structures. Unlike intersectionality, whose primary focus is on the intersection and the simultaneous impacts of social identities within the structures of power, most of the existing policies consider the determinants separately; they are also mainly focused on the socio-economic characteristics that prevent the identification of other dimensions of identity leading to inequalities [3, 5, 6].

Numerous studies have reviewed the application of intersectionality in the field of health. Given the evidence, intersectionality has been used more frequently because of its evolutionary and critical perspective toward identifying the causes of inequalities and

improving justice accordingly [7-9]. Although there is rich theoretical background in the basics of intersectionality, it is still necessary to be tested in the health domain [10]. Findings from our previous scoping review revealed that intersectionality had been employed inadequately pertaining to the design and implementation of health interventions [11]. Lack of familiarity of researchers and policymakers with this approach and its methodological challenges has led to its limited application [6, 11]. Intersectionality-based Policy Analysis Framework is considered a substantial effort to operationalize intersectionality. Hankivsky et al. introduced the nature, application, and also the principles of intersectionality including *"intersecting categories", "multilevel analysis", "power", "reflexivity", "time and space", "diverse knowledge", and "social justice & equity"* [3]. Although the development of this framework represents an essential step forward, it is still imperative to pursue further developments in order to emphasize methodological issues and their application in empirical studies [12].

Given the application of intersectionality and compliance with its principles, it is crucial to find out how intersectionality can be employed in the design and implementation of health programs. One of the global health issues that have been emphasized to end inequalities is HIV/AIDS [13]. The universal test-and-treat (UTT) strategy has been considered the cornerstone of the global AIDS control effort [14]. In spite of the substantial progress in this area, the progress to end HIV transmission is off track to reach the goal of ending AIDS "as a public health threat" by 2030. It is noteworthy that globally more than half of new HIV infections currently occur among key populations and their sexual partners. Besides, some groups are dealing with more AIDS- related deaths and complications as well as less access

to necessary interventions. However, most current HIV programs are not designed to meet the needs and realities of people living with these intersecting inequalities [13].

Immigrants are considered to be highly vulnerable to HIV/AIDS and are introduced as a key population in the 2025 AIDS targets [15]. According to the evidence, they are more likely to experience delay in HIV diagnosis and treatment compared to the host countries' population [16-20]. Moreover, different researches have investigated the effect of various individual, social, economic, and structural factors on immigrants' access to HIV services [17, 21, 22]. Hence, the international organizations have asked for efforts to reduce inequalities and help immigrant populations benefit from HIV services [23]. In this regards, we conducted a multi-stage study to apply intersectionality approach to design the peer intervention to promote the use of HIV testing among immigrants.

Materials and Methods:

To design a peer intervention through intersectionality approach to improve HIV testing uptake among Afghan immigrants in Iran, we conducted this study in five stages (Fig 1).

Fig 1. The steps of designing the peer intervention to improve the access of HIV testing among Afghan immigrants in Iran based on intersectionality approach

1- Qualitative study:

Given the emphasis of intersectionality on the importance of context in the development of experiences of oppression and privilege [4], we initially conducted a qualitative study between June 2018 and April 2020 to identify obstacles and also solutions regarding Afghan

immigrants' access to HIV services in the context of Iran. The details of the methodology are presented in our previous paper [24]. Briefly, the study participants included three groups of stakeholders: Afghan immigrants diagnosed with or at risk of HIV/AIDS (n=8), service providers (n=8), and policymakers/managers/experts (n=9). The participants were selected using purposive and snowball sampling. The data were then collected through semi-structured interviews. Data analysis was performed based on the content analysis method. We aimed to respect the principle of diverse knowledge in intersectionality, particularly the voice of the target group (immigrants), considering the opinions of different groups of stakeholders. Moreover, several factors were explored at different individual/interpersonal, organizational, and structural levels, in line with the principle of multilevel analysis in intersectionality approach [24]. Moreover, the lens of intersectionality was used to identify stigma and discrimination in accessing HIV services among Afghan immigrants [25].

2- Realist review:

By developing Context-Mechanism-Outcome Configurations (CMOCs) during the process of analysis and generating program theory, a realist review can identify how an intervention or action under certain contextual circumstances (C) may trigger a mechanism (M) to achieve a given outcome (O) [26,27]. Given the determinants and proposed solutions identified in our qualitative study, and based on our literature review, we decided to review the evidence about the peer interventions. Therefore, the realist review of peer interventions to improve HIV testing uptake among international immigrants was conducted; First, an initial program theory was developed regarding the mechanism of peer interventions and contextual factors. Then, this theory was tested through a review of evidence. The following inclusion criteria were considered for this realist review: different types of primary studies, interventional or

non-interventional studies with different designs investigating the international immigrant population and the effect of peers (as the provider of education, support, or counseling) on HIV testing uptake. The search resources included the following electronic databases: PubMed, Web of Science, Scopus, Embase, and Cochrane; the websites of related international organizations: The Joint United Nations Programmes on HIV/AIDS (UNAIDS), the World Bank, Global Fund, WHO, and International Organization for Migration (IOM); as well as the reference list of the related studies. After employing the Preferred Reporting Items for Systematic reviews and Meta-Analyses (PRISMA) process to select the relevant studies, the researchers conducted data extraction to explain the concepts of initial program theory and find potential relationships between them. In addition, the quality of the selected studies was evaluated. Two independent reviewers were assigned to conduct the steps of selecting studies, extracting data, and evaluating the quality of the evidence. They would also seek agreement in case of any disagreements. The content analysis method was used to analyze the data, and then strategies, intervention mechanisms, and influential contextual factors were identified during the data categorization. Furthermore, data coding and categorization procedures were discussed, and the consensus was achieved by the research team during several meetings. Eventually, the relationship between these concepts and different pathways was determined in theory and the refined program theory was presented (under review manuscript).

3- Adapting the contextual factors identified in the context of Iran and the realist review:

The uniformity of the obtained contextual factors in the qualitative study was evaluated with those identified in the realist review. For this purpose, the factors related to each theme (individual/interpersonal, service provision, and structural factors) from the qualitative study, as well as the contextual factors identified in the realist review, were classified and compared to examine the potential consistency of these contextual factors. Consequently, successful strategies in the evidence would be employed to design peer interventions to improve the use of HIV testing in the context of Iran.

4- Scoping review:

We also conducted a scoping review to determine health interventions designed and implemented based on the intersectionality approach. Based on that, a checklist was designed to evaluate the application of intersectionality in health interventions and programs, and it was also used in the present study. Details about the design of the checklist and its items have been published in our previous article [11]. In short, this checklist was prepared in order to facilitate the analysis of the existing health interventions and programs, as well as to help design, implement, and evaluate new interventions using the intersectionality approach by the health researchers, planners, and policymakers. After reviewing the literature for intersectionality in the field of social sciences and health, the items related to the principles of intersectionality were extracted according to different stages of health planning, namely identification of the problem, design & implementation, and evaluation. Various versions of the checklist were reviewed during several expert panels, and the required amendments were applied accordingly. Finally, a 38-item checklist was designed. In the present study, this checklist was used to moderate the considerations of observing the principles of intersectionality, which will be elaborated on in the next step.

5- Designing the Peer intervention to improve access to HIV testing services:

This phase was conducted according to the findings of the realist review based on successful intervention strategies, the findings of the qualitative study based on the factors and solutions facilitating immigrants' access to HIV testing, and the considerations of observing intersectionality principles extracted from the checklist developed in the scoping review study. Initially, the research team held several meetings on observing the intersectionality principles in the development and implementation of peer interventions. These discussion sessions were scheduled in order to help improve Afghan immigrants' access to HIV services and determine the considerations. In the next step, the researchers listed successful intervention strategies. Besides, they extracted the barriers identified in the qualitative study to determine the intervention components for each strategy. Then, they modified the components of the interventions so that they would be in line with intersectionality principles. Furthermore, the components should be adjusted to take into account the potential barriers and respective solutions according to each strategy. In other words, the strategies were presented consistent with intersectionality principles, available evidence, and the context.

Ethical considerations:

This study was reviewed and approved by the Ethics Committee of Tehran University of Medical Sciences, Iran (IR.TUMS.VCR.REC.1396.4171). We confirm that all methods were performed in accordance with the relevant guidelines and regulations and all of the processes approved with the institutional review board. The written informed consent was obtained

from all participants in the qualitative phase of the study. They were assured of confidentiality and anonymity about the information that they provided.

Results:

- ## Qualitative study: identifying barriers and proposing solutions regarding Afghan immigrants' access to HIV services in Iran

The findings of the qualitative study have been published in our previous article [24]. In total, 25 individuals participated in this study. The average age of immigrants was 40 years (range: 23-78). Three of the participating immigrant were female, and the rest were male. The immigrants had been living in Iran for 30.5 years on average within the range of 23 to 36 years. Moreover, most of them were married, undocumented, and the majority held low education degrees. They also reported poor working conditions, and most of them did not have any access to health insurance. The average age of service providers and policymakers was 38.37 years (range: 28-51) and 51.55 years (range: 39-74), respectively. Moreover, they held educational degrees in different disciplines. The factors influencing Afghan immigrants' access to HIV/AIDS services were categorized into 3 themes that were extracted from 9 categories: 1. Cultural (cultural similarities and differences, values and beliefs); 2. Psychosocial (social support, stigma and discrimination); and 3. Service delivery related factors (awareness, health services coverage and integrity, health services financing, accessibility, and continuity of care) [24]. According to the qualitative analysis via intersectionality lens, that group of immigrants who had language barriers in communication,

were in economic, social, cultural and supportive conditions, and had experienced stigma and discrimination, were more likely to confront problems with HIV testing services uptake. Such experiences were formed in the context of barriers at the level of service delivery, as well as the impact of immigration structures and regulations, educational policies, and poor intersectoral collaborations (Fig 2).

Fig 2. The intersection of factors influencing Afghan immigrants' access to HIV testing services

Furthermore, the participants proposed some solutions to improve immigrants' access to such services. These suggestions include raising HIV awareness (particularly regarding the nature of the disease, prevention, & diagnosis), providing financial and non-financial support, reducing stigma and discrimination at multiple levels, as well as providing outreach and peer-support services according to Afghan immigrants' needs and cultural values.

- ## Realist Review: Identifying intervention strategies and contextual factors to improve using HIV testing in immigrants

By developing the context-mechanism-outcome configurations during the process of analysis and generating a program theory, our realist review identified the peer interventions worked through four pathways: Following the improvement of communications (as a proximal mechanism): 1) increasing awareness, 2) reduced stigma, 3) improved support, and 4) increased access to services could lead to improved HIV testing uptake among immigrants. Peer interventions with multiple strategies (providing HIV information, providing support, community-based strategy, and providing HIV testing services) to be designed and

implemented considering the barriers to HIV testing and also moving beyond one-size-fits-all approaches can successfully improve the immigrants' HIV testing uptake. The providing HIV information strategy focuses on the implementation of different educational methods as well as the adaptation of educational content to immigrants' cultural conditions, educational needs, and existing barriers. Moreover, the "providing support" strategy highlights peers' role as supporters or advocators of immigrants through providing informational support and advice, contacting social network members, accompanying immigrants to HIV testing center, and defending their peers while experiencing potential violence. The community-based strategy encompasses different approaches such as the use of CBPR approaches and chain referral methods in studies, as well as providing outreach services. Regarding the providing HIV testing services strategy, peers usually play the role of someone who suggests referring to the testing center and performing HIV tests. In addition, contextual factors were categorized into three groups: individual/interpersonal factors (language, values , and beliefs, socio-economic status, immigration status, fear and stigma, gender), health service-related factors (accessibility, availability, acceptability, affordability), and structural factors (regulations and policies, supportive resources, discrimination).

- ## The results of adapting the contextual factors identified in the context of Iran and the realist review:

The comparison of the contextual factors affecting the use of HIV testing in the realist review of the evidence with the factors identified in our qualitative study indicated the similarities of the factors (Table.1).

Table 1. Contextual factors influencing HIV testing uptake extracted from evidence and context of Iran

Contextual factors	Realist review evidence	Qualitative study findings
**Intra/interpersonal factors**	Language and cultural barriers;<br>Poor knowledge about HIV and available services;<br>Poor economic and occupational conditions;<br>Illegal immigration and residential mobility;<br>Fear and stigma	Cultural factors (language, values, …);<br>poor knowledge about HIV and available services;<br>Low literacy levels;<br>Poor economic and occupational conditions;<br>Residential mobility;<br>Fear and stigma;<br>Poor social support
**Health service related factors**	Geographical accessibility issues;<br>Outreach services as the facilitator;<br>Limited working hours for HIV testing and long waiting time;<br>Providing services based on immigrants' needs;<br>Cultural and language conditions;<br>Peers' characteristics as the facilitator of service acceptability;<br>Affordability of services; poor economic conditions, lack of health insurance, and costly HIV testing	Service provision location (time and distance);<br>Improper provision of integrated services;<br>Lack of services based on immigrants' needs and characteristics;<br>Mistrust;<br>Lack of continuity of services due to uncertain residence issues and transfer of service providers;<br>Costs;<br>Occupational facilitators;<br>Free HIV tests;<br>Incentive for peers
**Structural factors**	Immigration regulations and fear of deportation<br>Criminalization of most aspects of sex work<br>Public health regulations of periodical HIV/STI testing for high-risk individuals as a facilitator<br>Insurance policies and costly HIV testing for immigrants<br>The facilitating role of media and NGOs, as well as the contribution of community-based organizations<br>Discrimination	Immigration regulations and fear of deportation<br><br>The facilitating role of providing free HIV testing to immigrants irrespective of their migration status<br>Poor educational policies<br>Improper intersectoral collaborations<br>The facilitating role of community participation (peers and NGOs)<br>Discrimination due to lack of antidiscrimination laws

- **Designing interventions to improve access to HIV testing services:**

It is critical to focus on different influential factors and their potential intersection while implementing intersectionality. Therefore, proposed interventions should include diverse strategies reflecting the barriers and their intersections as well as different individual, social, and structural levels. According to the findings of the realist review, the common features of successful interventions in improving HIV test uptake among immigrants included: 1) They used multiple strategies; 2) They were designed and implemented based on the immigrants' needs and conditions, and; 3) They have addressed the barriers of access to HIV testing services. Hence, we considered these features while designing the peer interventions. On the other hand, the checklist for the application of intersectionality in health interventions helped develop the list of considerations for intersectionality principles while designing peer interventions, which is presented in Box 1.

**Box 1. Considerations regarding intersectionality principles while designing and implementing peer intervention**
- The intervention/program should be designed and implemented based on intersection of immigrants' identities, as well as economic, social, and political conditions of the context. In other words, the interventions with multiple strategies should be designed to respond to intersectional needs/conditions affecting HIV testing uptake among immigrants.<br><br>- Interventions should be implemented at the different individual, organizational, and policy levels.<br><br>- According to the intersectionality principles, different groups of stakeholders are required to design and implement the intervention/program, including immigrants, service providers, and policymakers.<br><br>- It is necessary for HIV service providers and policy makers to recognize Afghan immigrants as a group with the most vulnerability and also to be informed about the cultural characteristics of immigrants.<br><br>- According to the intersectional stigma towards Afghan immigrants, the necessary conditions for maintaining the confidentiality of the immigrants' information should be considered in the design & implementation of the intervention, and required training should be provided to peers in this regard.<br><br>- The educational content, communication channels, and implementation methods (individual/group) should be conducted tailored to immigrants' conditions to increase knowledge and provide support to immigrants (flexible educational and support methods should be observed).

Lastly, the researchers determined the details of the proposed interventions to improve Afghan immigrants' HIV testing uptake. In the meantime, we attempted to observe the principles of intersectionality and the contextual factors identified in the qualitative study reflecting each strategy (Table 2).

Table 2. The interventions to improve taking HIV tests among Afghan immigrants in Iran

Intervention strategy	Intervention features	Identified contextual factors
**Providing information about HIV and available services**	- Provision of information to the immigrants by peers trained regarding HIV and existing services<br>- Provision of information based on the intersection between immigrants' social identities and structural conditions; employing a variety of educational approaches as well as designing the educational content in accordance with immigrants' needs and preferences (the instructional approaches need to be flexible).<br>- Raising HIV awareness in the society as well as the service providers	Cultural factors (language barriers, values, and beliefs)<br>Low literacy level<br>Poor HIV awareness<br>Fear and stigma
**Providing support**	- Improving financial and non-financial support<br>- Involving community-based support resources<br>- Educating peers on supporting skills and familiarizing them with available support resources	Poor economic and occupational conditions<br>Poor social support<br>Discrimination
**Providing community-based testing services**	- Providing continuous HIV counseling and testing services<br>- Observing confidentiality issues while providing services to minimize the stigma<br>- Providing accessible HIV counseling and testing services to immigrants (outreach, door-to-door, and etc.)<br>- Including various groups of stakeholders, such as immigrants, service providers, community-based organizations, media, and policymakers<br>- Providing HIV counseling and testing services based on immigrants'	Infrequent provision of services due to immigrants' residential mobility and potential transfer of service providers in the healthcare system<br>Stigma and discrimination<br><br>Location (time and distance)

	preferences, needs, and conditions<br>- Providing services by peers<br>- Familiarizing employees with immigrants’ culture and conditions to build trust	Providing services irrespective of immigrants’ needs and conditions<br>Lack of trust to health system
**Structural/policy-making interventions**	- Promoting public HIV awareness<br>- Implementing necessary measures to avoid discrimination<br>- Acknowledging immigrants’ status<br>- Improving intersectoral collaborations in the provision of HIV services (media, educational system, community-based organizations, etc.)	Poor educational policies<br>Discrimination in service provision due to lack of antidiscrimination laws<br>The facilitating role of free HIV testing services to immigrants irrespective of their migration conditions<br>Migration regulations and fear of deportation<br>The facilitating role of community participation (peers and NGOs)<br>Discrimination<br>Poor intersectoral collaboration

Discussion:

The present study highlighted the applicability of intersectionality through designing peer intervention, aiming to improve the use of HIV testing among Afghan immigrants in Iran. The researchers intended to assess the context through reviewing of the evidence as well as exploring stakeholders’ perspectives, and address the factors at different levels. As an example of the application of intersectionality in the field of health, this study can be helpful in enriching our knowledge regarding the application of intersectionality in practice.

Oppressed and marginalized people are regarded as one of the significant tenets of intersectionality concerning public health [5]. In this regard, the present study has focused on

Afghan immigrants as a vulnerable and marginalized group in Iran. Afghan immigrants, as the largest group of immigrants in Iran, typically suffer from poor economic, social, and occupational conditions; besides, previous systematic reviews have indicated their deprivation of social and health services [28, 29]. The findings of our qualitative study revealed that the majority of these immigrants reported low literacy levels as well as inappropriate economic and employment status. They argued that most of them did not have a valid migration card and health insurance; furthermore, they faced different obstacles accessing HIV services [24].

Challenging the existing power structures and valuing the voices of vulnerable groups are emphasized in the intersectionality approach [3]. Accordingly, the present study aimed to listen to the immigrants' voices, balance the power, and pay attention to the power structures through following ways:

Firstly, the researchers aimed to identify barriers and existing structures regarding the context of HIV services in Iran from the perspective of several stakeholders, and at different individual, organizational, and policy levels before designing the intervention. Meanwhile, the intersectionality principles of “diverse knowledge”, “multilevel analysis”, and “power” were addressed. Besides, identifying the immigrants' lived experiences about accessing HIV services could particularly contribute to pay attention to their voices. The relationship between power and knowledge production is highlighted within the intersectionality approach. In order to reduce the power pressure in health planning or policies, it is crucial to consider the perspectives and worldviews of marginalized groups who are exclude from the knowledge production process [30]. Furthermore, our realist review was employed to examine the evidence to identify the strategies of successful peer interventions, in line with

the principle of “diverse knowledge”. It was also intended to pay attention to the contextual factors affecting the efficiency of the interventions in the existing evidence. These strategies were then used in designing the intervention after approving the adaptation of this evidence to the context of Iran.

Secondly, the "*peer*" interventions were selected in accordance with the principles of intersectionality. Hence, in the context where there are discriminatory structures, especially towards HIV and Afghan immigrants, the findings of the realist review highlighted that the interventions through the participation of peers, who were among the target group with some similarities in terms of social identities, could lead to a power balance that might play a critical role in the success of the interventions. Peers’ ability to communicate with immigrants on an equal level by understanding their challenges can help the success of these peer interventions. Equal relationships put peers in a position where they can play the role of a mediator between the client and the service provider, depending on the needs and the existing situation so as to develop the balance in power [31]. On the other hand, one of the performance characteristics of peers is encouraging the target populations in participatory learning processes, and empowering them. Since empowerment is crucial in reducing health inequalities and accessing their health care services, such interventions target marginalized and socially disadvantaged groups [32]. Moreover, the supportive peer relationship can provide a more critical understanding of how personal experience is related to social and political struggles and actively promote “social justice” [32]. It seems that it is necessary to pay more attention to the value of the peers’ role. Moreover, it is suggested to define systematically their position, especially in the context of health systems with limited resources.

Thirdly, we proposed intervention strategies at individual levels (raising awareness, providing support, & involving the target group in providing testing services) as well as structural interventions at policy levels. Therefore, it was recommended to promote HIV-related educational policies, implement discrimination prevention guidelines, acknowledge immigrants at the policy level, and promote intersectoral collaboration while providing HIV services. One might hope to reduce inequalities in practice by proposing changes at the policy levels; similarly, disregarding this issue and designing health interventions at individual levels has been mentioned as one of the obstacles to the successful implementation of existing policies in reducing inequalities. On the other hand, the intersectionality perspective has a significant potential to reduce inequalities by emphasizing the identification and challenging power structures [6]. In intersectionality-based studies, interventions should focus on systems and power structures, including health care systems, public policy, regulations, education, and economy, rather than highlighting individuals and groups [33]. In this regard, it has been confirmed that in advocacy-based peer interventions, behavior changes at the individual level or health outcomes are possible only if changes occur at the levels of social power structures [32].The findings of our previous scoping review also indicated that the impacts of power structures, such as policies in emphasizing or responding to the problem are neglected in most studies [11].

Given the limited application of intersectionality in health interventions, it is essential to employ the instruments and processes to apply this approach step by step, from problem identification to designing and implementing the intervention. It can further help familiarize researchers and policymakers with this issue and operationalize this approach in the design and implementation of health programs. Meanwhile, it is recommended to observe the

intersectionality principles. The following considerations would facilitate the operationalization of intersectionality: the participation of diverse groups of stakeholders, especially the target group; highlighting the intersection of conditions at different micro, meso, and macro levels; the identification of power structures; and arranging evidence-based strategies for interventions in accordance with the context conditions as well as the intersection of target population' identities.

Strengths and limitations of the study:

Provided that the application of intersectionality is associated with various challenges, this study intended to illustrate the operational use of intersectionality by reporting a case of designing peer interventions aimed to improve the use of HIV testing in a group of immigrants; it also seems that health planners can employ this approach to design interventions through intersectionality approach. According to the evidence, it is necessary to conduct further research on the enhancement of designs and methods that can effectively include the principles of intersectionality [34]. In this regard, a checklist was employed in the present study to evaluate the application of intersectionality in different stages of health planning. This checklist can be implemented as an objective instrument to observe the principles of intersectionality in the design, implementation, and evaluation of interventions.

It is noteworthy that the preliminary study conducted to obtain first-hand data on the barriers and respective solutions for immigrants to access HIV services was merely qualitative. Nevertheless, the use of mixed method approaches is emphasized in evaluating the impact of inequalities on the life experiences of marginalized people [35, 36]. It seems that such approaches should be employed to collect, analyze, and integrate quantitative data with

qualitative data in order to perform a more accurate evaluation of the intersection of social identities and power structures with the aim of identifying the characteristics of the most vulnerable people, and helping policymakers in prioritizing and allocating resources. Therefore, it is recommended to use the mixed method approach in the process of designing health interventions.

Conclusions:

Intersectionality has been increasingly used by researchers as an emerging approach in the field of health. However, the practical application of intersectionality is still regarded as a research gap and requires further research in this field. The present study highlighted the applicability of intersectionality in the design of a health intervention by providing a practical framework for researchers and health planners who are concerned with reducing health inequalities. Accordingly, it is necessary to pay attention to the relationships and structures of the context, determine the intervention components based on the evidence, needs of the target group, as well as apply changes at different levels.

Acknowledgments:

We thank Professor Reza Negarandeh, Professor AbouAli Vedadhir , Professor Amirhossein Takian, Professor Abolghasem Pourreza, Professor Elham Shakibazadeh, and Professor Miziar Moradi-Lakeh. Their consultations were very helpful in improving the quality of our study.

References:

1. Gkiouleka A, Huijts T, Beckfield J, Bambra C. Understanding the micro and macro politics of health: Inequalities, intersectionality & institutions-A research agenda. Social Science & Medicine. 2018;200:92-8.

2. Heard E, Fitzgerald L, Wigginton B, Mutch A. Applying intersectionality theory in health promotion research and practice. Health Promotion International. 2020;35(4):866-876.

3. Hankivsky O, Grace D, Hunting G. An intersectionality-based policy analysis framework. Vancouver, BC: Institute for Intersectionality Research and Policy, Simon Fraser University. 2012.

4. Kelly C, Kasperavicius D, Duncan D, Etherington C, Giangregorio L, Presseau J, et al. ‘Doing’or ‘using’ intersectionality? Opportunities and challenges in incorporating intersectionality into knowledge translation theory and practice. International Journal for Equity in Health. 2021;20(1):1-7.

5. Bowleg L. The problem with the phrase women and minorities: intersectionality—an important theoretical framework for public health. American journal of public health. 2012;102(7):1267-73.

6. Holman D, Salway S, Bell A, Beach B, Adebajo A, Ali N, et al. Can intersectionality help with understanding and tackling health inequalities? Perspectives of professional stakeholders. medRxiv. 2020.

7. Couto MT, Oliveira Ed, Separavich MAA, Luiz OdC. The feminist perspective of intersectionality in the field of public health: a narrative review of the theoretical-methodological literature. Salud Colectiva. 2019;15:e1994.

8. Harari L, Lee C. Intersectionality in quantitative health disparities research: A systematic review of challenges and limitations in empirical studies. Social Science & Medicine. 2021:113876.

9. Larson E, George A, Morgan R, Poteat T. 10 Best resources on… intersectionality with an emphasis on low-and middle-income countries. Health policy and planning. 2016;31(8):964-9.

10. Green MA, Evans CR, Subramanian SV. Can intersectionality theory enrich population health research? Soc Sci Med. 2017;178:214-216.

11. Ghasemi E, Majdzadeh R, Rajabi F, Vedadhir A, Negarandeh R, Jamshidi E, et al. Applying Intersectionality in designing and implementing health interventions: a scoping review. BMC public health. 2021;21(1):1-13.

12. Holman D, Salway S, Bell A, Beach B, Adebajo A, Ali N, et al. Can intersectionality help with understanding and tackling health inequalities? Perspectives of professional stakeholders. Health research policy and systems. 2021;19(1):1-15.

13. UNAIDS. Global AIDS strategy 2021-2026. END INEQUALITIES. END AIDS. 2021. Available at: https://www.unaids.org/sites/default/files/media_asset/global-AIDS-strategy-2021-2026_en.pdf

14. Sanders EJ, Agutu CA, Graham SM. Multiple HIV testing strategies are necessary to end AIDS. AIDS (London, England). 2021;35(12):2039.

15. UNAIDS. 2025 AIDS targets. 2021. Available at: https://www.unaids.org/sites/default/files/2025-AIDS-Targets_en.pdf

16. Alvarez-del Arco D, Monge S, Azcoaga A, Rio I, Hernando V, Gonzalez C, et al. HIV testing and counselling for migrant populations living in high-income countries: a systematic review. The European Journal of Public Health. 2013;23(6):1039-45.

17. Blondell SJ, Kitter B, Griffin MP, Durham J. Barriers and facilitators to HIV testing in migrants in high-income countries: a systematic review. AIDS and Behavior. 2015;19(11):2012-24.

18. Keygnaert I, Guieu A, Ooms G, Vettenburg N, Temmerman M, Roelens K. Sexual and reproductive health of migrants: Does the EU care? Health policy. 2014;114(2-3):215-25.

19. Kronfli N, Linthwaite B, Sheehan N, Cox J, Hardy I, Lebouché B, et al. Delayed linkage to HIV care among asylum seekers in Quebec, Canada. BMC public health. 2019;19(1):1-9.

20. Pottie K, Lotfi T, Kilzar L, Howeiss P, Rizk N, Akl EA, et al. The effectiveness and cost-effectiveness of screening for HIV in migrants in the EU/EEA: a systematic review. International journal of environmental research and public health. 2018;15(8):1700.

21. Fakoya I, Álvarez-del Arco D, Copas AJ, Teixeira B, Block K, Gennotte A-F, et al. Factors associated with access to HIV testing and primary care among migrants living in Europe: cross-sectional survey. JMIR public health and surveillance. 2017;3(4):e7741.

22. Suphanchaimat R, Sommanustweechai A, Khitdee C, Thaichinda C, Kantamaturapoj K, Leelahavarong P, et al. HIV/AIDS health care challenges for cross-country migrants in low-and middle-income countries: a scoping review. Hiv/aids (Auckland, NZ). 2014;6:19.

23. International Organization for Migration. Human mobility and HIV, 2020. Available at: https://www.iom.int/human-mobility-hiv

24. Ghasemi, E., Rajabi, F., Majdzadeh, R., Vedadhir, A., Negarandeh, R. Aspects Influencing Access to HIV/AIDS Services among Afghan Immigrants in Iran: A Qualitative Study. *International Journal of Community Based Nursing & Midwifery*, 2022; 10(3): 172-183.

25. Ghasemi E, Rajabi F, Negarandeh R, Vedadhir A, Majdzadeh R. HIV, migration, gender, and drug addiction: A qualitative study of intersectional stigma towards Afghan immigrants in Iran. Health Soc Care Community. 2022 Sep;30(5):e1917-e1925.

26. Pawson R, Greenhalgh T, Harvey G, Walshe K. Realist review-a new method of systematic review designed for complex policy interventions. J Health Serv Res Policy. 2005;10(1_suppl):21-34.

27. Wong G, Westhorp G, Pawson R, Greenhalgh T. Realist synthesis. RAMESES training materials London: The RAMESES Project. 2013. Available at: https://www.ramesesproject.org/media/Realist_reviews_training_materials.pdf

28. Gooshki ES, Rezaei R, Wild V. Migrants' Health in Iran from the Perspective of Social Justice: a Systematic Literature Review. Archives of Iranian Medicine (AIM). 2016;19(10).

29. Roozbeh N, Sanati A, Abdi F. Afghan Refugees and Immigrants Health Status in Iran: A Systematic Review. Journal of Clinical & Diagnostic Research. 2018;12(9).

30. Hankivsky O. Intersectionality 101. The Institute for Intersectionality Research & Policy, SFU. 2014;36.

31. MacLellan J, Surey J, Abubakar I, Stagg HR. Peer support workers in health: a qualitative metasynthesis of their experiences. PloS one. 2015;10(10):e0141122.

32. Simoni JM, Franks JC, Lehavot K, Yard SS. Peer interventions to promote health: conceptual considerations. American Journal of Orthopsychiatry. 2011;81(3):351.

33. Kelly UA. Integrating intersectionality and biomedicine in health disparities research. Advances in Nursing Science. 2009;32(2):E42-E56.

34. Hancock A-M. When multiplication doesn't equal quick addition: Examining intersectionality as a research paradigm. Perspectives on politics. 2007;5(1):63-79.

35. Agénor M. Future directions for incorporating intersectionality into quantitative population health research. American journal of public health. 2020;110(6):803-6.

36. Bowleg L, Bauer G. Invited reflection: Quantifying intersectionality. Psychology of Women Quarterly. 2016;40(3):337-41.

Fig 1 Click here to access/download;Figure;Fig 1.tif

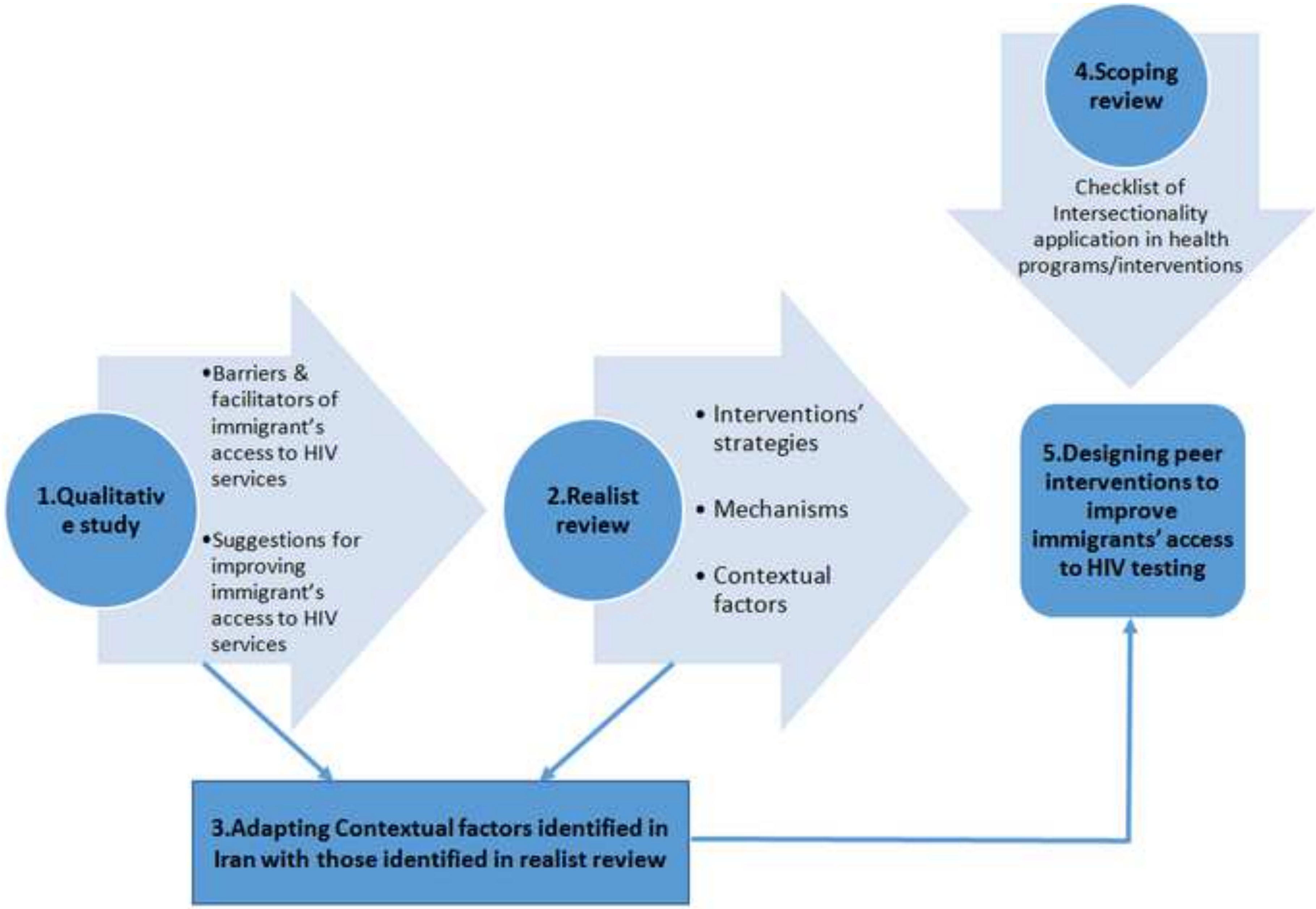

Fig 2

Click here to access/download;Figure;Fig 2.tif

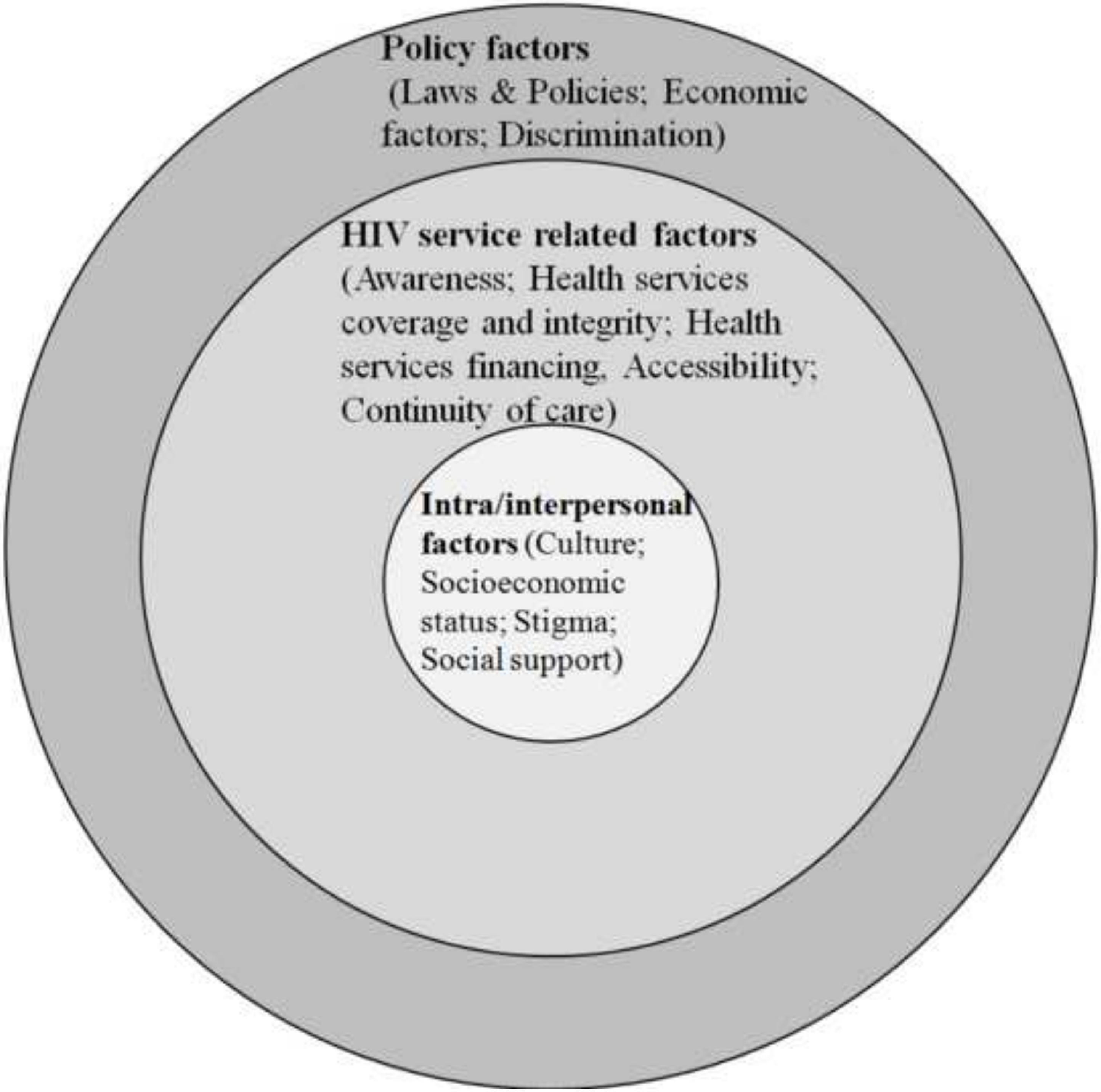

Supplement: Attachment — Submitted filename: PONE-D-23-10867_reviewer.pdf [file pone.0335311.s002.pdf]
