# Supplementary material for: From theory to practice of designing for diversity: Applying intersectionality to improve HIV testing uptake
Source: PLoS One. 2025 Oct 27;20(10):e0335311. doi: 10.1371/journal.pone.0335311 (PMC12558453; doi:10.1371/journal.pone.0335311)
Supplement: Attachment — Submitted filename: PLOS one study comments.docx [file pone.0335311.s001.docx]

General comments

The methodology of the project seems complex and rigorous, which is a strength. However, given that each stage of the study has its own methods and subsequent results, the flow of the paper is constantly interrupted, which can be confusing. Perhaps at the end of the methods section, the author could write a brief paragraph to tie the different moving parts together?

A few grammatical errors were noted:

- Page 10 “Three of the participating immigrants were female”
- Page 18 “it is crucial to consider the perspectives and worldviews of marginalized groups who are excluded from the knowledge production process”

Specific comments

Introduction:

- Great introduction of intersectionality. Although the term was coined by Crenshaw, it is important to note that the concept itself is a product of Black feminist scholarship and had been used by other scholars beforehand.
- The authors did a good job introducing why immigrants are the target population. However, it would have been helpful to get more insight on the specific disparities in HIV prevalence and/or risk among Afghan immigrants in Iran.
  - This was briefly done in the discussion section, but having some of this information in the background section would orient the reader better to the authors’ selection of study site and population.

Methods:

- The summary of the qualitative section is well done. It would be helpful for the authors to add more detail on the following:
  - Who conducted the semi-structured interviews with different stakeholders?
  - How exactly was intersectionality operationalized in the qualitative portion of the process? This question can be answered by providing more detail in this sentence: “Moreover, several factors were explored at different individual/interpersonal, organizational, and structural levels, in line with the principle of multilevel analysis in intersectionality approach” [page 6]
    - See phase 5 for reference

Results:

- The first paragraph is clear; however, more detail is needed on how the different social identities individuals held hindered their ability to access HIV services. This could be an extra sentence towards the end of the paragraph, and would segue well into Fig 2.
- The section on designing interventions (page 14) includes some of the author’s interpretation. The first two sentences can be moved to the discussion.

Discussion:

- The flow is often interrupted by the author’s chosen connectives; perhaps replace some of the connectives with punctuation marks that serve a similar role.
- Designing a qualitative study is NOT a limitation. Reference to page 21: “It is noteworthy that the preliminary study conducted to obtain first-hand data on the barriers and respective solutions for immigrants to access HIV services was merely qualitative.”
