# Supplementary material for: From theory to practice of designing for diversity: Applying intersectionality to improve HIV testing uptake
Source: PLoS One. 2025 Oct 27;20(10):e0335311. doi: 10.1371/journal.pone.0335311 (PMC12558453; doi:10.1371/journal.pone.0335311)
Supplement: Attachment — Submitted filename: Reveiw Feedback_Reza et al.docx [file pone.0335311.s003.docx]

**ABSTRACT**

**Introduction**: This section was well written.

**Methodology**:

- It does not describe study design – exploratory? Descriptive? Explanatory?
- What research method did you use - Quantitative? Qualitative? Mixed method which could either be mixed qualitative, mixed quantitative or both? These must be clearly stated.
- You should have highlighted your sampling technique and sample size. You must consider your readers who would stop at abstract and ensure your paper is compelling enough to move your reader to the next section
- You did not demonstrate how you controlled confidentiality and consent even though you have a section ethical

**Results/Conclusion**

- Did application of intersectionality improve HIV testing uptake or not? This is missing out in the results and conclusions. Hence, making findings and conclusions divergent from the topic of discission. In other words, results and conclusions are no in line with study objectives which aims to operationalize intersectionality and conducted a multi-stage study proposing interventions to seek improvement in taking HIV test among Afghan immigrants in Iran

**MAIN MANUSCRIPT**

**Method**

- Follow my comments in the abstract and provide details in this section.
- In the scoping review section, schematically illustrate how many articles retrieved, reviewed and included, criteria for exclusion etc.
- See annotations in the attached PDF

**Additional comments**

- He manuscript requires Englis language proof reading before it can be accepted it’s form
- Overall, this is an interesting study
