# Supplementary material for: From theory to practice of designing for diversity: Applying intersectionality to improve HIV testing uptake
Source: PLoS One. 2025 Oct 27;20(10):e0335311. doi: 10.1371/journal.pone.0335311 (PMC12558453; doi:10.1371/journal.pone.0335311)
Supplement: Attachment — Submitted filename: Response to reviewers.docx [file pone.0335311.s004.docx]

Authors’ response to the review of manuscript entitled **“From theory to practice of designing for diversity: applying intersectionality to improve HIV testing uptake”**

| **Academic Editor’s Comments:** | **Authors’ response** |
| --- | --- |
| This study intends to make use of the potential benefits of intersectionality and its intention is to apply the framework of intersectionality to inform the development of an intervention to increase HIV testing. However, there are some major flaws in the manuscript. There is some confusion in terms of what the paper focuses on; it is unclear if the paper is reporting on the findings of a study or whether this manuscript is a process paper describing how an intervention was developed using the framework of intersectionality. As an example – the abstract describes in the introduction that this is a multi-stage study but then talks about a peer-led intervention in the results section. There was no mention of a peer-led intervention in the introduction of the abstract.  Please consider that the paper is reporting on how an intervention is developed – and make use of the following guidelines: Guidance for reporting intervention development studies in health research (GUIDED). | We are thankful for the insightful review of this manuscript. In the revised manuscript, we mentioned that this is an intervention development study and we used intersectionality approach for the design of a peer-led intervention guided by the 2008 the Medical Research Council (MRC). Accordingly, necessary corrections were made in the abstract and main text. As your comment, we used GUIDED to report our manuscript according the following reference:  Duncan E, O'Cathain A, Rousseau N, Croot L, Sworn K, Turner KM, Yardley L, Hoddinott P. Guidance for reporting intervention development studies in health research (GUIDED): an evidence-based consensus study. BMJ Open. 2020;10(4):e033516.  The corrections are done with track changes.  -According to the journal requirements, the corrections were done (Funding Statement, Data availability statement, and uploading figures to the PACE).  -It is notable that the data related to each part of the conducted studies has been published in our previous articles; therefore, in order to consider publication ethics, we have not supporting information file in the present study. In order to provide supporting information for readers, we have cited to these articles in the text of the article as appropriate. We added this explanation to Data availability statement. |
| Please check in-text referencing throughout the paper – a few examples in the Introduction – there were no references linked to the text: In spite of the substantial progress in this area, the progress to end HIV transmission is off track to reach the goal of ending AIDS “as a public health threat” by 2030. It is noteworthy that globally more than half of new HIV infections currently occur among key populations and their sexual partners. Besides, some groups are dealing with more AIDS- related deaths and complications as well as less access | We added the reference in the text. |
| Methods – • Please discuss the limitations inherent in this small sample of the qualitative study. Please can you also elaborate why the qualitative part of this study took two years?  • Please can you provide more information regarding the scoping review? Was it published? What were the major findings and how are those findings incorporated in the development of the intervention is not very clear. | -In qualitative studies, sampling is done until data saturation. In the present study, during the process of content analysis, data saturation occurred up to our sample size. The long duration of the qualitative study was due to the sensitivity of the issue (HIV/AIDS) and the time-consuming process of obtaining permission to conduct the study. In addition, most of the Afghan immigrants in Iran are undocumented, and this made it difficult to reach them.  -More information regarding scoping review was added in the methods and results sections. In the text, we mentioned that the scoping review study has been published in our previous article. [Reference No.12]. (Materials & Methods Section, Stage1: Identifying existing evidence, at the end of paragraph) |
| Overall spelling and grammar need to be checked throughout the manuscript | The entire manuscript is revised accordingly. |
| For Lab, Study and Registered Report Protocols: These article types are not expected to include results but may include pilot data. | Not applicable |
| **Reviewer(1)' Comments:** |  |
| General comments  The methodology of the project seems complex and rigorous, which is a strength. However, given that each stage of the study has its own methods and subsequent results, the flow of the paper is constantly interrupted, which can be confusing. Perhaps at the end of the methods section, the author could write a brief paragraph to tie the different moving parts together?  A few grammatical errors were noted:   - Page 10 “Three of the participating immigrants were female” - Page 18 “it is crucial to consider the perspectives and worldviews of marginalized groups who are excluded from the knowledge production process” | -By applying the Medical Research Council (MRC) framework, as well as using Guidance for reporting intervention development studies in health research (GUIDED), as a standard tool to report the methodology and results, we tried to rewrite the manuscript clearly.  -These grammatical errors were corrected. |
| Specific comments  Introduction:   - Great introduction of intersectionality. Although the term was coined by Crenshaw, it is important to note that the concept itself is a product of Black feminist scholarship and had been used by other scholars beforehand. - The authors did a good job introducing why immigrants are the target population. However, it would have been helpful to get more insight on the specific disparities in HIV prevalence and/or risk among Afghan immigrants in Iran.   - This was briefly done in the discussion section, but having some of this information in the background section would orient the reader better to the authors’ selection of study site and population. | -Thank you for your comment. We added related information in Introduction section, lines 6-11.  This information was mentioned at the end of Introduction section. |
| Methods:   - The summary of the qualitative section is well done. It would be helpful for the authors to add more detail on the following:   - Who conducted the semi-structured interviews with different stakeholders?   - How exactly was intersectionality operationalized in the qualitative portion of the process? This question can be answered by providing more detail in this sentence: “Moreover, several factors were explored at different individual/interpersonal, organizational, and structural levels, in line with the principle of multilevel analysis in intersectionality approach” [page 6]     - See phase 5 for reference | -We have added the information about who conducted the semi-structured interviews (Materials & Methods Section, stage 3: Modelling process and outcomes, Paragraph 2.)  - The information about the application of intersectionality in the qualitative study was added (Materials & Methods Section, stage 3: Modelling process and outcomes, Paragraph 2.)  -Phase 5 was removed. |
| Results:   - The first paragraph is clear; however, more detail is needed on how the different social identities individuals held hindered their ability to access HIV services. This could be an extra sentence towards the end of the paragraph, and would segue well into Fig 2. - The section on designing interventions (page 14) includes some of the author’s interpretation. The first two sentences can be moved to the discussion. | We added more explanation at the end of the paragraph. In the revised version, this part is located in the Results section; the stage 3; the modeling process and outcomes; the results of the qualitative study.  - These sentences were removed from the Results section. |
| Discussion:   - The flow is often interrupted by the author’s chosen connectives; perhaps replace some of the connectives with punctuation marks that serve a similar role. - Designing a qualitative study is NOT a limitation. Reference to page 21: “It is noteworthy that the preliminary study conducted to obtain first-hand data on the barriers and respective solutions for immigrants to access HIV services was merely qualitative.” | -It was tried to improve the coherence and sequence of discussion by adding related sentences.  - Corrections were made in the section of “Strengths and limitations of the study”. During the revising of the manuscript, this sentence was removed. |
| **Reviewer(2)' Comments:** |  |
| **ABSTRACT**  **Introduction**: This section was well written.  **Methodology**:   - It does not describe study design – exploratory? Descriptive? Explanatory? - What research method did you use - Quantitative? Qualitative? Mixed method which could either be mixed qualitative, mixed quantitative or both? These must be clearly stated. - You should have highlighted your sampling technique and sample size. You must consider your readers who would stop at abstract and ensure your paper is compelling enough to move your reader to the next section - You did not demonstrate how you controlled confidentiality and consent even though you have a section ethical   **Results/Conclusion**   - Did application of intersectionality improve HIV testing uptake or not? This is missing out in the results and conclusions. Hence, making findings and conclusions divergent from the topic of discussion. In other words, results and conclusions are no in line with study objectives which aims to operationalize intersectionality and conducted a multi-stage study proposing interventions to seek improvement in taking HIV test among Afghan immigrants in Iran | **Methodology**:  -In the revised manuscript, we mentioned that this is an intervention development study and we used intersectionality approach for the design of a peer-led intervention guided by the 2008 the Medical Research Council (MRC) framework. Accordingly, necessary corrections were made in the abstract and main text.  -According the comment about ensuring the confidentiality and consent, more explanations were added in the section of Ethical consideration, at the end of Methods section.  **Results/Conclusion**  - In this study, we did not aim to evaluate the effectiveness of the peer intervention to improve HIV testing uptake. Instead, we focused on the method of application of intersectionality to design the intervention. In the revised manuscript, we used the intervention development step from the 2008 MRC framework as a guide to conduct the study, and according to the purpose of the study, the MRC stages that are related to the intervention evaluation were not used. In this regard, the sections of the Method, Results, Discussion and Conclusions have been focused on the application of intersectionality in the study. |
| **MAIN MANUSCRIPT**  **Method**   - Follow my comments in the abstract and provide details in this section. - In the scoping review section, schematically illustrate how many articles retrieved, reviewed and included, criteria for exclusion etc. - See annotations in the attached PDF   **Additional comments**   - The manuscript requires English language proof reading before it can be accepted it’s form - Overall, this is an interesting study | -In the revised manuscript, we mentioned that this is an intervention development study and we used intersectionality approach for the design of a peer-led intervention guided by the 2008 the Medical Research Council (MRC) framework. Accordingly, necessary corrections were made in the abstract and main text.  -More information regarding scoping review was added in the methods and results sections. In the text, we mentioned this study has been published in our previous article. [Reference No.12]. (Materials & Methods Section, Stage1: Identifying existing evidence, at the end of paragraph). PRISMA flowchart has been shown in our published paper (Reference no. 12).  The manuscript was reviewed by an English language expert and respective modifications are applied. |
