# Supplementary material for: From theory to practice of designing for diversity: Applying intersectionality to improve HIV testing uptake
Source: PLoS One. 2025 Oct 27;20(10):e0335311. doi: 10.1371/journal.pone.0335311 (PMC12558453; doi:10.1371/journal.pone.0335311)
Supplement: Attachment — Submitted filename: Response to reviewers 2.docx [file pone.0335311.s005.docx]

Authors’ response to the review of manuscript entitled **“From theory to practice of designing for diversity: applying intersectionality to improve HIV testing uptake”**

Reviewers' comments:

Reviewer's Responses to Questions

**Comments to the Author**

1. If the authors have adequately addressed your comments raised in a previous round of review and you feel that this manuscript is now acceptable for publication, you may indicate that here to bypass the “Comments to the Author” section, enter your conflict of interest statement in the “Confidential to Editor” section, and submit your "Accept" recommendation.

Reviewer #1: (No Response)

Reviewer #3: (No Response)

2. Is the manuscript technically sounds, and do the data support the conclusions?

The manuscript must describe a technically sound piece of scientific research with data that supports the conclusions. Experiments must have been conducted rigorously, with appropriate controls, replication, and sample sizes. The conclusions must be drawn appropriately based on the data presented.

Reviewer #1: Yes

Reviewer #3: No

3. Has the statistical analysis been performed appropriately and rigorously?

Reviewer #1: N/A

Reviewer #3: N/A

4. Have the authors made all data underlying the findings in their manuscript fully available?

The [PLOS Data policy](https://linkprotect.cudasvc.com/url?a=http%3a%2f%2fwww.plosone.org%2fstatic%2fpolicies.action%23sharing&c=E,1,WB81BO6wMPVkJ_3n7ZGTlrRnyBUrmBE66qltGEYkH6pte1A3-YpxHIAihemDW5ZqPdi0C1L0zUm2VwOwrvoqNS4uv39LqA5Ck4D-fsHfoie1hHV3oLrSfg,,&typo=1) requires authors to make all data underlying the findings described in their manuscript fully available without restriction, with rare exception (please refer to the Data Availability Statement in the manuscript PDF file). The data should be provided as part of the manuscript or its supporting information, or deposited to a public repository. For example, in addition to summary statistics, the data points behind means, medians and variance measures should be available. If there are restrictions on publicly sharing data—e.g. participant privacy or use of data from a third party—those must be specified.

Reviewer #1: Yes

Reviewer #3: (No Response)

5. Is the manuscript presented in an intelligible fashion and written in standard English?

PLOS ONE does not copyedit accepted manuscripts, so the language in submitted articles must be clear, correct, and unambiguous. Any typographical or grammatical errors should be corrected at revision, so please note any specific errors here.

Reviewer #1: Yes

Reviewer #3: Yes

**6. Review Comments to the Author:**

Please use the space provided to explain your answers to the questions above. You may also include additional comments for the author, including concerns about dual publication, research ethics, or publication ethics. (Please upload your review as an attachment if it exceeds 20,000 characters)

Reviewer #1: The author did a good job addressing the feedback from reviewers. The chosen framework was well-supported and operationalized. It is a good addition to the literature on addressing health inequities.

Authors’ response:

We are thankful for the insightful review of this manuscript.

Reviewer #3: This is an interesting manuscript of how intersectionality can lead the development of interventions. However, there are some major methodological gaps.

Authors claim that "This is an intervention development study. We used the intersectionality approach to design a peer-led intervention guided by the 2008 MRC framework" however, I do not think this is an intervention development study, mainly because readers cannot see that intervention. I would say that they identify major categories based on intersectionality that should be addressed in interventions on the specified phenomenon. But this is not an intervention development, there is no an intervention in this manuscript (sessions, components, facilitators, structure of the intervention).

The aim of the study was how to design an intervention that has the necessary characteristics to comply with the principles of intersectionality. According to the editor and reviewers’ comments in the first step of manuscript review, we used the MRC framework in order to carry out this process in a structured manner. In this regard, we presented the study method in detail according to the steps of the framework. According to the the UHC Compendium, as a global repository of interventions for UHC, interventions are generally defined and described based on categories of health programme, population, life course stage, resources required, delivery platform (WHO, 2022). In this regard, we reported the intervention features for each of the intervention pathways by addressing intersectionality principles while designing peer intervention in the stage of modeling process and outcomes.

-Reference: World Health Organization. UHC compendium. health interventions for universal health coverage. Version 1.3. 2022.‏ Available at: <https://www.who.int/universal-health-coverage/compendium#:~:text=The%20UHC%20Compendium%20is%20a,health%20services%20and%20health%20interventions>

I recommend to name the Combahee River Collective as one of the main referents of black feminism, and Hill-Collins and Bowleg as the most recent and important referents of intersectionality.

We added the referents of intersectionality mentioned by the reviewer (Introduction section, Page 3).

Methodology needs clarification. For example, it looks like authors developed two different reviews in phase 1 and 2. But the scoping review in phase 1 is not well explained. Then, phase 2 include criteria that is not clear, why did authors decide to include "interventional or non-interventional studies with different designs investigating the international immigrant population". Based on intersectionality, is it assumed that all international immigrant face the same accessing problems? (human rights, health)?

This is a very interesting manuscript but the lack of clarity makes it hard to read and to understand which is the actual goal. If this is an intersectionality based study, some criteria for evidence retrieval may rise concern.

According the comment about explanation of the scoping review, more information was added in the section of “Materials & Methods- Identifying existing evidence”.

As methodological sources of realist reviews have explained (Pawson et al, 2005; Wong et al, 2013), data relevant to a realist synthesis may lie in a broad range of sources that may cross traditional disciplinary, program, and sector boundaries. The search phase is thus likely to involve searching for different sorts of data, or studies from different domains, with which to test different aspects of the provisional theory. Realist syntheses do not exclude sources solely on the basis of their study design; hence, ‘methodological filters’ (e.g. to identify randomised controlled trials) add little to the search and run the risk of excluding relevant papers.

Based on key tenets of intersectionality; “the importance of any category or structure cannot be predetermined; the categories and their importance must be discovered in the process of investigation” (Hankivsky, 2014). So, researchers might hinder identifying the intersection of categories in all groups by focusing on *a specific/sub group of immigrants*. On the other hand, this is not in line with the principle of reflexivity. So, through an exploratory intersectionality lens, we decided to select all international immigrants as population criteria to review the evidence. Then, in the process of intervention development, the target population for intervention is determined by using the results of qualitative study and explores stakeholders’ perspectives on immigrants’ needs and circumstances and other contextual factors.

-Pawson R, Greenhalgh T, Harvey G, Walshe K. Realist review-a new method of systematic review designed for complex policy interventions. *J Health Serv Res Policy* (2005) 10(1_suppl):21-34.

-Wong G, Westhorp G, Pawson R, Greenhalgh T. Realist synthesis. RAMESES training materials London: The RAMESES Project (2013) Available at: <https://www.ramesesproject.org/media/Realist_reviews_training_materials.pdf>

-Hankivsky O. Intersectionality 101. The Institute for Intersectionality Research & Policy, SFU. 2014;36.

7. PLOS authors have the option to publish the peer review history of their article ([what does this mean?](https://linkprotect.cudasvc.com/url?a=https%3a%2f%2fjournals.plos.org%2fplosone%2fs%2feditorial-and-peer-review-process%23loc-peer-review-history&c=E,1,L8CxUg9M1lStc5rAkdZw2R1EzcOIlrDsQwKx1pjVeer4LoMJKJbQv-mBuQ00trzcUv-_lMmqq9V9XzHq0_vsq7nc3DjgowePJkBpaFVvJd-eVA,,&typo=1)). If published, this will include your full peer review and any attached files.


If you choose “no”, your identity will remain anonymous but your review may still be made public.


**Do you want your identity to be public for this peer review?** For information about this choice, including consent withdrawal, please see our [Privacy Policy](https://linkprotect.cudasvc.com/url?a=https%3a%2f%2fwww.plos.org%2fprivacy-policy&c=E,1,I-WtDR0GL590KJHHNQTFeaUHukAfmwCs6KH_QCqOouovtDjDQr5fTbPiTm7YqsSJQU0SRRgLrz7lzWzkaZxBsvdhxgmkcfiJu8GM_VDYTyPqsZA,&typo=1).

Reviewer #1: No

Reviewer #3: **Yes:** A. A. Villa-Rueda
