# Supplementary material for: From theory to practice of designing for diversity: Applying intersectionality to improve HIV testing uptake
Source: PLoS One. 2025 Oct 27;20(10):e0335311. doi: 10.1371/journal.pone.0335311 (PMC12558453; doi:10.1371/journal.pone.0335311)
Supplement: Attachment — Submitted filename: Response to reviewers 3.docx [file pone.0335311.s006.docx]

Authors’ response to the review of manuscript entitled **“From theory to practice of designing for diversity: applying intersectionality to improve HIV testing uptake”**

Reviewers' comments:

Reviewer's Responses to Questions

**Comments to the Author**

1. If the authors have adequately addressed your comments raised in a previous round of review and you feel that this manuscript is now acceptable for publication, you may indicate that here to bypass the “Comments to the Author” section, enter your conflict of interest statement in the “Confidential to Editor” section, and submit your "Accept" recommendation.

Reviewer #1: All comments have been addressed

Reviewer #3: (No Response)

2. Is the manuscript technically sound, and do the data support the conclusions?

The manuscript must describe a technically sound piece of scientific research with data that supports the conclusions. Experiments must have been conducted rigorously, with appropriate controls, replication, and sample sizes. The conclusions must be drawn appropriately based on the data presented.

Reviewer #1: Yes

Reviewer #3: Partly

3. Has the statistical analysis been performed appropriately and rigorously?

Reviewer #1: Yes

Reviewer #3: N/A

4. Have the authors made all data underlying the findings in their manuscript fully available?

The [PLOS Data policy](https://linkprotect.cudasvc.com/url?a=http%3a%2f%2fc05y1x9s.r.us-east-2.awstrack.me%2fL0%2fhttp%3a%252F%252Fwww.plosone.org%252Fstatic%252Fpolicies.action%2523sharing%2f1%2f010f0191bad67368-888a437e-9a77-4406-a6af-5f9e2780688e-000000%2f9LgHhxIqK1nOfgC7_cMuJXbHbwc%3d175&c=E,1,HkhtaTIpeSXZmPmYGdP5qjLmvVmNWo-Bbr3PVj0XjF10qqspSLCJEoQQ-AbuuSltVrKL8aj9Mu-AN18llRTtO2oPAgcfYzZJ5320xxFBajAc2HmG33FOTVW8Zw,,&typo=1) requires authors to make all data underlying the findings described in their manuscript fully available without restriction, with rare exception (please refer to the Data Availability Statement in the manuscript PDF file). The data should be provided as part of the manuscript or its supporting information, or deposited to a public repository. For example, in addition to summary statistics, the data points behind means, medians and variance measures should be available. If there are restrictions on publicly sharing data—e.g. participant privacy or use of data from a third party—those must be specified.

Reviewer #1: Yes

Reviewer #3: No

5. Is the manuscript presented in an intelligible fashion and written in standard English?

PLOS ONE does not copyedit accepted manuscripts, so the language in submitted articles must be clear, correct, and unambiguous. Any typographical or grammatical errors should be corrected at revision, so please note any specific errors here.

Reviewer #1: Yes

Reviewer #3: Yes

6. Review Comments to the Author

Please use the space provided to explain your answers to the questions above. You may also include additional comments for the author, including concerns about dual publication, research ethics, or publication ethics. (Please upload your review as an attachment if it exceeds 20,000 characters)

Reviewer #1: I went through the manuscript and the tracked changes document and the authors have responded adequately to my comments.

Reviewer #3: The article is now clearer; authors did a good work adding the recommendations.
However, there are some methodological parts that could still be improved, especially with the intention of being replicable. The abstract and the aim of the study still need further clarification.

**Authors’ response:**

We are thankful for the insightful review of this manuscript. For more clarification, explanations about the method and the purpose of the study were added. The corrections are done with track changes.

In the abstract:

**Introduction:** Given the critical perspective of intersectionality and its potential to identify the causes of inequalities, it has been employed increasingly in studies related to health. Despite the rich theoretical evidence about intersectionality, there is a need to consider this approach empirically. This study aimed to apply the intersectionality in practice for health policy makers and researchers seeking health inequalities. In this regard, we described the development of an intersectionality-based and context-specific intervention focusing on HIV testing uptake among Afghan immigrants in Iran.

**Methods:** This is an intervention development study. The intersectionality was used to design a peer-led intervention guided by the 2008 MRC framework. We undertook the following activities related to the three stages of the MRC framework: 1. Identifying the existing evidence (conducting a scoping review to investigate the application of intersectionality in designing and implementing health interventions; designing the checklist of applying the intersectionality in health interventions and programs) ; 2. Identifying and developing a program theory (conducting a realist review to identify why, how, and under what conditions peer interventions can improve HIV testing uptake among immigrants); and 3. Modeling process and outcomes (adapting the contextual factors identified by conducting a qualitative study and the realist review; extracting considerations regarding intersectionality principles using the checklist of applying the intersectionality in health interventions and programs; determining context specific, intersectionality-based and evidence-based intervention components for each of the intervention pathways).

**Results:** According to considerations regarding checklist of application of intersectionality principles, the intervention at the different individual, organizational, and policy levels with multiple strategies should be designed to respond to needs/conditions affecting HIV testing uptake among immigrants. We determined the peer-led intervention features to improve the use of HIV testing services in Afghan immigrants following the intersectionality principles, target group needs, and contextual conditions aiming to modify power structures. Intervention strategies included HIV information provision, support, community-based services, and structural interventions.

7. PLOS authors have the option to publish the peer review history of their article ([what does this mean?](https://linkprotect.cudasvc.com/url?a=https%3a%2f%2fc05y1x9s.r.us-east-2.awstrack.me%2fL0%2fhttps%3a%252F%252Fjournals.plos.org%252Fplosone%252Fs%252Feditorial-and-peer-review-process%2523loc-peer-review-history%2f1%2f010f0191bad67368-888a437e-9a77-4406-a6af-5f9e2780688e-000000%2fZcWGdhwJUM9Lz760LGE8RyViV8U%3d175&c=E,1,MBaQfDYoE32X8b7mjabEkzmuTpZtZiLDZAkX6M2jAvEQGRmNu4ub90pdrFJF9XiPk9W7I0a0ihEx9ZjvgKvHI4qf_dQAfSoT0uIcwXu-oFd-fA,,&typo=1)). If published, this will include your full peer review and any attached files.


If you choose “no”, your identity will remain anonymous but your review may still be made public.


**Do you want your identity to be public for this peer review?** For information about this choice, including consent withdrawal, please see our [Privacy Policy](https://linkprotect.cudasvc.com/url?a=https%3a%2f%2fc05y1x9s.r.us-east-2.awstrack.me%2fL0%2fhttps%3a%252F%252Fwww.plos.org%252Fprivacy-policy%2f1%2f010f0191bad67368-888a437e-9a77-4406-a6af-5f9e2780688e-000000%2fTMesmxNFk-HSP3GkRExiv7EpeN4%3d175&c=E,1,DZLT7IifSvWbrwPo5DNJ0Hwko8dTZ3leDS3lYKwbtEk6dcYJrR_u8QB5cmt9sORtKgtrZ3wudGz8qFL73p9MYCUrJUfh-2bneOFuPfKwMMsQNAU,&typo=1).

Reviewer #1: No

Reviewer #3: No
