# Supplementary material for: From theory to practice of designing for diversity: Applying intersectionality to improve HIV testing uptake
Source: PLoS One. 2025 Oct 27;20(10):e0335311. doi: 10.1371/journal.pone.0335311 (PMC12558453; doi:10.1371/journal.pone.0335311)
Supplement: Peer review history

# Peer review: Decision Letter 0

**DOI:** 10.1371/journal.pone.0335311.r002

**Authors:** Allanise Cloete-Mapingire

11 Oct 2023

Dear Dr. Majdzadeh,

Thank you for submitting your manuscript to PLOS ONE. After careful consideration, we feel that it has merit but does not fully meet PLOS ONE’s publication criteria as it currently stands. Therefore, we invite you to submit a revised version of the manuscript that addresses the points raised during the review process.

==============================

Please consider that the paper is reporting on how an intervention is developed – and make use of the following guidelines: Guidance for reporting intervention development studies in health research (GUIDED).

Please check in-text referencing throughout the paper – a few examples in the Introduction – there were no references linked to the text:

In spite of the substantial progress in this area, the progress to end HIV transmission is off track to reach the goal of ending AIDS “as a public health threat” by 2030. It is noteworthy that globally more than half of new HIV infections currently occur among key populations and their sexual partners. Besides, some groups are dealing with more AIDS- related deaths and complications as well as less access

Methods –

• Please discuss the limitations inherent in this small sample of the qualitative study. Please can you also elaborate why the qualitative part of this study took two years?

• Please can you provide more information regarding the scoping review? Was it published? What were the major findings and how are those findings incorporated in the development of the intervention is not very clear.

Overall spelling and grammar need to be checked throughout the manuscript

For Lab, Study and Registered Report Protocols: These article types are not expected to include results but may include pilot data. 

==============================

Please submit your revised manuscript by Nov 25 2023 11:59PM. If you will need more time than this to complete your revisions, please reply to this message or contact the journal office at plosone@plos.org . When you're ready to submit your revision, log on to https://www.editorialmanager.com/pone/ and select the 'Submissions Needing Revision' folder to locate your manuscript file.

- A rebuttal letter that responds to each point raised by the academic editor and reviewer(s). You should upload this letter as a separate file labeled 'Response to Reviewers'.
- A marked-up copy of your manuscript that highlights changes made to the original version. You should upload this as a separate file labeled 'Revised Manuscript with Track Changes'.
- An unmarked version of your revised paper without tracked changes. You should upload this as a separate file labeled 'Manuscript'.

If you would like to make changes to your financial disclosure, please include your updated statement in your cover letter. Guidelines for resubmitting your figure files are available below the reviewer comments at the end of this letter.

If applicable, we recommend that you deposit your laboratory protocols in protocols.io to enhance the reproducibility of your results. Protocols.io assigns your protocol its own identifier (DOI) so that it can be cited independently in the future. For instructions see: https://journals.plos.org/plosone/s/submission-guidelines#loc-laboratory-protocols . Additionally, PLOS ONE offers an option for publishing peer-reviewed Lab Protocol articles, which describe protocols hosted on protocols.io. Read more information on sharing protocols at https://plos.org/protocols?utm_medium=editorial-email&utm_source=authorletters&utm_campaign=protocols .

We look forward to receiving your revised manuscript.

Kind regards,

Allanise Cloete, PhD (Anthropology)

Academic Editor

PLOS ONE

Journal Requirements:

When submitting your revision, we need you to address these additional requirements.

1. Please ensure that your manuscript meets PLOS ONE's style requirements, including those for file naming. The PLOS ONE style templates can be found at

https://journals.plos.org/plosone/s/file?id=wjVg/PLOSOne_formatting_sample_main_body.pdf and

https://journals.plos.org/plosone/s/file?id=ba62/PLOSOne_formatting_sample_title_authors_affiliations.pdf .

2. Thank you for stating in your Funding Statement:

“This work was supported by Tehran University of Medical Sciences (TUMS), Grant number [96-03-62-36567]. The funder had no role in study design, data collection and analysis, decision to publish, or preparation of the manuscript.”

Please provide an amended statement that declares *all* the funding or sources of support (whether external or internal to your organization) received during this study, as detailed online in our guide for authors at http://journals.plos.org/plosone/s/submit-now.  Please also include the statement “There was no additional external funding received for this study.” in your updated Funding Statement.

Please include your amended Funding Statement within your cover letter. We will change the online submission form on your behalf.

3. In your Data Availability statement, you have not specified where the minimal data set underlying the results described in your manuscript can be found. PLOS defines a study's minimal data set as the underlying data used to reach the conclusions drawn in the manuscript and any additional data required to replicate the reported study findings in their entirety. All PLOS journals require that the minimal data set be made fully available. For more information about our data policy, please see http://journals.plos.org/plosone/s/data-availability.

"Upon re-submitting your revised manuscript, please upload your study’s minimal underlying data set as either Supporting Information files or to a stable, public repository and include the relevant URLs, DOIs, or accession numbers within your revised cover letter. For a list of acceptable repositories, please see http://journals.plos.org/plosone/s/data-availability#loc-recommended-repositories. Any potentially identifying patient information must be fully anonymized.

Important: If there are ethical or legal restrictions to sharing your data publicly, please explain these restrictions in detail. Please see our guidelines for more information on what we consider unacceptable restrictions to publicly sharing data: http://journals.plos.org/plosone/s/data-availability#loc-unacceptable-data-access-restrictions. Note that it is not acceptable for the authors to be the sole named individuals responsible for ensuring data access.

We will update your Data Availability statement to reflect the information you provide in your cover letter.

[Note: HTML markup is below. Please do not edit.]

Reviewers' comments:

Reviewer's Responses to Questions

**Comments to the Author**

1. Is the manuscript technically sound, and do the data support the conclusions?

Reviewer #1: Yes

Reviewer #2: Yes

**********

2. Has the statistical analysis been performed appropriately and rigorously?

Reviewer #1: Yes

Reviewer #2: Yes

**********

3. Have the authors made all data underlying the findings in their manuscript fully available?

The PLOS Data policy

Reviewer #1: Yes

Reviewer #2: Yes

**********

4. Is the manuscript presented in an intelligible fashion and written in standard English?

Reviewer #1: Yes

Reviewer #2: No

**********

Reviewer #1: Please see attached file

Reviewer #2: ABSTRACT

Introduction: This section was well written.

Methodology:

• It does not describe study design – exploratory? Descriptive? Explanatory?

• What research method did you use - Quantitative? Qualitative? Mixed method which could either be mixed qualitative, mixed quantitative or both? These must be clearly stated.

• You should have highlighted your sampling technique and sample size. You must consider your readers who would stop at abstract and ensure your paper is compelling enough to move your reader to the next section

• You did not demonstrate how you controlled confidentiality and consent even though you have a section ethical

Results/Conclusion

• Did application of intersectionality improve HIV testing uptake or not? This is missing out in the results and conclusions. Hence, making findings and conclusions divergent from the topic of discission. In other words, results and conclusions are no in line with study objectives which aims to operationalize intersectionality and conducted a multi-stage study proposing interventions to seek improvement in taking HIV test among Afghan immigrants in Iran

MAIN MANUSCRIPT

Method

• Follow my comments in the abstract and provide details in this section.

• In the scoping review section, schematically illustrate how many articles retrieved, reviewed and included, criteria for exclusion etc.

• See annotations in the attached PDF

Additional comments

• He manuscript requires English language proof reading before it can be accepted it’s form

• Overall, this is an interesting study

**********

what does this mean? ). If published, this will include your full peer review and any attached files.

If you choose “no”, your identity will remain anonymous but your review may still be made public.

**Do you want your identity to be public for this peer review?** For information about this choice, including consent withdrawal, please see our Privacy Policy

Reviewer #1: No

Reviewer #2: No

**********

[NOTE: If reviewer comments were submitted as an attachment file, they will be attached to this email and accessible via the submission site. Please log into your account, locate the manuscript record, and check for the action link "View Attachments". If this link does not appear, there are no attachment files.]

While revising your submission, please upload your figure files to the Preflight Analysis and Conversion Engine (PACE) digital diagnostic tool, https://pacev2.apexcovantage.com/ . PACE helps ensure that figures meet PLOS requirements. To use PACE, you must first register as a user. Registration is free. Then, login and navigate to the UPLOAD tab, where you will find detailed instructions on how to use the tool. If you encounter any issues or have any questions when using PACE, please email PLOS at figures@plos.org . Please note that Supporting Information files do not need this step.

Attachment Submitted filename: PLOS one study comments.docx

Attachment Submitted filename: PONE-D-23-10867_reviewer.pdf

Attachment Submitted filename: Reveiw Feedback_Reza et al.docx

# Author comment: Author response to Decision Letter 1

**DOI:** 10.1371/journal.pone.0335311.r003

11 Mar 2024

We have responded to each point raised by the academic editor and reviewers and uploaded responses as a separate file labeled 'Response to Reviewers'.

Attachment Submitted filename: Response to reviewers.docx

# Peer review: Decision Letter 1

**DOI:** 10.1371/journal.pone.0335311.r004

**Authors:** Allanise Cloete-Mapingire

3 Jun 2024

Dear Dr. Majdzadeh,

Thank you for submitting your manuscript to PLOS ONE. After careful consideration, we feel that it has merit but does not fully meet PLOS ONE’s publication criteria as it currently stands. Therefore, we invite you to submit a revised version of the manuscript that addresses the points raised during the review process.

Please submit your revised manuscript by Jul 18 2024 11:59PM. If you will need more time than this to complete your revisions, please reply to this message or contact the journal office at plosone@plos.org . When you're ready to submit your revision, log on to https://www.editorialmanager.com/pone/ and select the 'Submissions Needing Revision' folder to locate your manuscript file.

- A rebuttal letter that responds to each point raised by the academic editor and reviewer(s). You should upload this letter as a separate file labeled 'Response to Reviewers'.
- A marked-up copy of your manuscript that highlights changes made to the original version. You should upload this as a separate file labeled 'Revised Manuscript with Track Changes'.
- An unmarked version of your revised paper without tracked changes. You should upload this as a separate file labeled 'Manuscript'.

If you would like to make changes to your financial disclosure, please include your updated statement in your cover letter. Guidelines for resubmitting your figure files are available below the reviewer comments at the end of this letter.

If applicable, we recommend that you deposit your laboratory protocols in protocols.io to enhance the reproducibility of your results. Protocols.io assigns your protocol its own identifier (DOI) so that it can be cited independently in the future. For instructions see: https://journals.plos.org/plosone/s/submission-guidelines#loc-laboratory-protocols . Additionally, PLOS ONE offers an option for publishing peer-reviewed Lab Protocol articles, which describe protocols hosted on protocols.io. Read more information on sharing protocols at https://plos.org/protocols?utm_medium=editorial-email&utm_source=authorletters&utm_campaign=protocols .

We look forward to receiving your revised manuscript.

Kind regards,

Allanise Cloete, PhD (Anthropology)

Academic Editor

PLOS ONE

[Note: HTML markup is below. Please do not edit.]

Reviewers' comments:

Reviewer's Responses to Questions

**Comments to the Author**

Reviewer #1: (No Response)

Reviewer #3: (No Response)

**********

2. Is the manuscript technically sound, and do the data support the conclusions?

Reviewer #1: Yes

Reviewer #3: No

**********

3. Has the statistical analysis been performed appropriately and rigorously?

Reviewer #1: N/A

Reviewer #3: N/A

**********

4. Have the authors made all data underlying the findings in their manuscript fully available?

The PLOS Data policy

Reviewer #1: Yes

Reviewer #3: (No Response)

**********

5. Is the manuscript presented in an intelligible fashion and written in standard English?

Reviewer #1: Yes

Reviewer #3: Yes

**********

Reviewer #1: The author did a good job addressing the feedback from reviewers. The chosen framework was well-supported and operationalized. It is a good addition to the literature on addressing health inequities

Reviewer #3: This is a interesting manuscript of how intersectionality can lead the development of interventions. However, there are some major methodological gaps.

Authors claim that "This is an intervention development study. We used the intersectionality approach to design a peer-led intervention guided by the 2008 MRC framework" however, I do not think this is an intervention development study, mainly because readers cannot see that intervention. I would say that they identify major categories based on intersectionality that should be addressed in interventions on the specified phenomenon. But this is not an intervention development, there is no an intervention in this manuscript (sessions, components, facilitators, structure of the intervention).

I recommend to name the Combahee River Collective as one of the main referents of black feminism, and Hill-Collins and Bowleg as the most recent and important referents of intersectionality.

Methodology needs clarification. For example, it looks like authors developed two different reviews in phase 1 and 2. But the scoping review in phase 1 is not well explained. Then, phase 2 include criteria that is not clear, why did authors decide to include "interventional or non-interventional studies with different designs investigating the international immigrant population". Based on intersectionality, is it assumed that all international immigrant face the same accessing problems? (human rights, health)?

This is a very interesting manuscript but the lack of clarity makes it hard to read and to understand which is the actual goal. If this is an intersectionality based study, some criteria for evidence retrieval may rise concern.

**********

what does this mean? ). If published, this will include your full peer review and any attached files.

If you choose “no”, your identity will remain anonymous but your review may still be made public.

**Do you want your identity to be public for this peer review?** For information about this choice, including consent withdrawal, please see our Privacy Policy

Reviewer #1: No

Reviewer #3: **Yes: ** A. A. Villa-Rueda

**********

[NOTE: If reviewer comments were submitted as an attachment file, they will be attached to this email and accessible via the submission site. Please log into your account, locate the manuscript record, and check for the action link "View Attachments". If this link does not appear, there are no attachment files.]

While revising your submission, please upload your figure files to the Preflight Analysis and Conversion Engine (PACE) digital diagnostic tool, https://pacev2.apexcovantage.com/ . PACE helps ensure that figures meet PLOS requirements. To use PACE, you must first register as a user. Registration is free. Then, login and navigate to the UPLOAD tab, where you will find detailed instructions on how to use the tool. If you encounter any issues or have any questions when using PACE, please email PLOS at figures@plos.org . Please note that Supporting Information files do not need this step.

# Author comment: Author response to Decision Letter 2

**DOI:** 10.1371/journal.pone.0335311.r005

26 Jun 2024

Authors’ response to the review of manuscript entitled “From theory to practice of designing for diversity: applying intersectionality to improve HIV testing uptake”

Reviewers' comments:

Reviewer's Responses to Questions

Comments to the Author

1. If the authors have adequately addressed your comments raised in a previous round of review and you feel that this manuscript is now acceptable for publication, you may indicate that here to bypass the “Comments to the Author” section, enter your conflict of interest statement in the “Confidential to Editor” section, and submit your "Accept" recommendation.

Reviewer #1: (No Response)

Reviewer #3: (No Response)

________________________________________

2. Is the manuscript technically sounds, and do the data support the conclusions?

The manuscript must describe a technically sound piece of scientific research with data that supports the conclusions. Experiments must have been conducted rigorously, with appropriate controls, replication, and sample sizes. The conclusions must be drawn appropriately based on the data presented.

Reviewer #1: Yes

Reviewer #3: No

________________________________________

3. Has the statistical analysis been performed appropriately and rigorously?

Reviewer #1: N/A

Reviewer #3: N/A

________________________________________

4. Have the authors made all data underlying the findings in their manuscript fully available?

The PLOS Data policy requires authors to make all data underlying the findings described in their manuscript fully available without restriction, with rare exception (please refer to the Data Availability Statement in the manuscript PDF file). The data should be provided as part of the manuscript or its supporting information, or deposited to a public repository. For example, in addition to summary statistics, the data points behind means, medians and variance measures should be available. If there are restrictions on publicly sharing data—e.g. participant privacy or use of data from a third party—those must be specified.

Reviewer #1: Yes

Reviewer #3: (No Response)

________________________________________

5. Is the manuscript presented in an intelligible fashion and written in standard English?

PLOS ONE does not copyedit accepted manuscripts, so the language in submitted articles must be clear, correct, and unambiguous. Any typographical or grammatical errors should be corrected at revision, so please note any specific errors here.

Reviewer #1: Yes

Reviewer #3: Yes

________________________________________

6. Review Comments to the Author:

Please use the space provided to explain your answers to the questions above. You may also include additional comments for the author, including concerns about dual publication, research ethics, or publication ethics. (Please upload your review as an attachment if it exceeds 20,000 characters)

Reviewer #1: The author did a good job addressing the feedback from reviewers. The chosen framework was well-supported and operationalized. It is a good addition to the literature on addressing health inequities.

Authors’ response:

We are thankful for the insightful review of this manuscript.

Reviewer #3:

This is an interesting manuscript of how intersectionality can lead the development of interventions. However, there are some major methodological gaps.

Authors claim that "This is an intervention development study. We used the intersectionality approach to design a peer-led intervention guided by the 2008 MRC framework" however, I do not think this is an intervention development study, mainly because readers cannot see that intervention. I would say that they identify major categories based on intersectionality that should be addressed in interventions on the specified phenomenon. But this is not an intervention development, there is no an intervention in this manuscript (sessions, components, facilitators, structure of the intervention).

Authors’ response:

The aim of the study was how to design an intervention that has the necessary characteristics to comply with the principles of intersectionality. According to the editor and reviewers’ comments in the first step of manuscript review, we used the MRC framework in order to carry out this process in a structured manner. In this regard, we presented the study method in detail according to the steps of the framework. According to the the UHC Compendium, as a global repository of interventions for UHC, interventions are generally defined and described based on categories of health programme, population, life course stage, resources required, delivery platform (WHO, 2022). In this regard, we reported the intervention features for each of the intervention pathways by addressing intersectionality principles while designing peer intervention in the stage of modeling process and outcomes.

-Reference: World Health Organization. UHC compendium. health interventions for universal health coverage. Version 1.3. 2022.‏ Available at: https://www.who.int/universal-health-coverage/compendium#:~:text=The%20UHC%20Compendium%20is%20a,health%20services%20and%20health%20interventions

I recommend to name the Combahee River Collective as one of the main referents of black feminism, and Hill-Collins and Bowleg as the most recent and important referents of intersectionality.

Authors’ response:

We added the referents of intersectionality mentioned by the reviewer (Introduction section, Page 3).

Methodology needs clarification. For example, it looks like authors developed two different reviews in phase 1 and 2. But the scoping review in phase 1 is not well explained. Then, phase 2 include criteria that is not clear, why did authors decide to include "interventional or non-interventional studies with different designs investigating the international immigrant population". Based on intersectionality, is it assumed that all international immigrant face the same accessing problems? (human rights, health)?

This is a very interesting manuscript but the lack of clarity makes it hard to read and to understand which is the actual goal. If this is an intersectionality based study, some criteria for evidence retrieval may rise concern.

Authors’ response:

According the comment about explanation of the scoping review, more information was added in the section of “Materials & Methods- Identifying existing evidence”.

As methodological sources of realist reviews have explained (Pawson et al, 2005; Wong et al, 2013), data relevant to a realist synthesis may lie in a broad range of sources that may cross traditional disciplinary, program, and sector boundaries. The search phase is thus likely to involve searching for different sorts of data, or studies from different domains, with which to test different aspects of the provisional theory. Realist syntheses do not exclude sources solely on the basis of their study design; hence, ‘methodological filters’ (e.g. to identify randomised controlled trials) add little to the search and run the risk of excluding relevant papers.

Based on key tenets of intersectionality; “the importance of any category or structure cannot be predetermined; the categories and their importance must be discovered in the process of investigation” (Hankivsky, 2014). So, researchers might hinder identifying the intersection of categories in all groups by focusing on a specific/sub group of immigrants. On the other hand, this is not in line with the principle of reflexivity. So, through an exploratory intersectionality lens, we decided to select all international immigrants as population criteria to review the evidence. Then, in the process of intervention development, the target population for intervention is determined by using the results of qualitative study and explores stakeholders’ perspectives on immigrants’ needs and circumstances and other contextual factors.

-Pawson R, Greenhalgh T, Harvey G, Walshe K. Realist review-a new method of systematic review designed for complex policy interventions. J Health Serv Res Policy (2005) 10(1_suppl):21-34.

-Wong G, Westhorp G, Pawson R, Greenhalgh T. Realist synthesis. RAMESES training materials London: The RAMESES Project (2013) Available at: https://www.ramesesproject.org/media/Realist_reviews_training_materials.pdf

-Hankivsky O. Intersectionality 101. The Institute for Intersectionality Research & Policy, SFU. 2014;36.

________________________________________

7. PLOS authors have the option to publish the peer review history of their article (what does this mean?). If published, this will include your full peer review and any attached files.

If you choose “no”, your identity will remain anonymous but your review may still be made public.

Do you want your identity to be public for this peer review? For information about this choice, including consent withdrawal, please see our Privacy Policy.

Reviewer #1: No

Reviewer #3: Yes: A. A. Villa-Rueda

Attachment Submitted filename: Response to reviewers 2.docx

# Peer review: Decision Letter 2

**DOI:** 10.1371/journal.pone.0335311.r006

**Authors:** Emily Hotez

3 Sep 2024

Dear Dr. Majdzadeh,

Thank you for submitting your manuscript to PLOS ONE. After careful consideration, we feel that it has merit but does not fully meet PLOS ONE’s publication criteria as it currently stands. Therefore, we invite you to submit a revised version of the manuscript that addresses the points raised during the review process.

Please submit your revised manuscript by Oct 18 2024 11:59PM. If you will need more time than this to complete your revisions, please reply to this message or contact the journal office at plosone@plos.org . When you're ready to submit your revision, log on to https://www.editorialmanager.com/pone/ and select the 'Submissions Needing Revision' folder to locate your manuscript file.

- A rebuttal letter that responds to each point raised by the academic editor and reviewer(s). You should upload this letter as a separate file labeled 'Response to Reviewers'.
- A marked-up copy of your manuscript that highlights changes made to the original version. You should upload this as a separate file labeled 'Revised Manuscript with Track Changes'.
- An unmarked version of your revised paper without tracked changes. You should upload this as a separate file labeled 'Manuscript'.

If you would like to make changes to your financial disclosure, please include your updated statement in your cover letter. Guidelines for resubmitting your figure files are available below the reviewer comments at the end of this letter.

If applicable, we recommend that you deposit your laboratory protocols in protocols.io to enhance the reproducibility of your results. Protocols.io assigns your protocol its own identifier (DOI) so that it can be cited independently in the future. For instructions see: https://journals.plos.org/plosone/s/submission-guidelines#loc-laboratory-protocols . Additionally, PLOS ONE offers an option for publishing peer-reviewed Lab Protocol articles, which describe protocols hosted on protocols.io. Read more information on sharing protocols at https://plos.org/protocols?utm_medium=editorial-email&utm_source=authorletters&utm_campaign=protocols .

We look forward to receiving your revised manuscript.

Kind regards,

Emily Hotez

Academic Editor

PLOS ONE

Additional Editor Comments:

Dear Author,

Thank you for the opportunity to review “From theory to practice of designing for diversity: applying intersectionality to improve HIV testing uptake.” This article aimed to describe the development of an intersectionality-based and context-specific intervention focusing on HIV testing uptake among Afghan immigrants in Iran. This study provides a practical framework for health planners and researchers seeking to reduce inequalities by presenting how intersectionality can influence the design of a health intervention. This aspect of the manuscript addresses an important gap in the literature—that is, that intersectionality is often discussed theoretically but not clearly articulated from an empirical perspective.

Below are some key strengths of this manuscript.

-The literature review on intersectionality is strong and includes a nuanced perspective of different aspects of intersectionality principles.

-The activities utilized to apply intersectionality towards developing a health intervention are clearly and appropriately described.

-It is notable that one phase of this work involved a full scoping review and embedded qualitative study that generated key insights.

-The authors were responsive to reviewers’ comments and requested revisions.

I look forward to seeing this article accepted in PLOS One.

[Note: HTML markup is below. Please do not edit.]

Reviewers' comments:

Reviewer's Responses to Questions

**Comments to the Author**

Reviewer #1: All comments have been addressed

Reviewer #3: (No Response)

**********

2. Is the manuscript technically sound, and do the data support the conclusions?

Reviewer #1: Yes

Reviewer #3: Partly

**********

3. Has the statistical analysis been performed appropriately and rigorously?

Reviewer #1: Yes

Reviewer #3: N/A

**********

4. Have the authors made all data underlying the findings in their manuscript fully available?

The PLOS Data policy

Reviewer #1: Yes

Reviewer #3: No

**********

5. Is the manuscript presented in an intelligible fashion and written in standard English?

Reviewer #1: Yes

Reviewer #3: Yes

**********

Reviewer #1: I went through the manuscript and the tracked changes document and the authors have responded adequately to my comments.

Reviewer #3: The article is now clearer, authors did a good work adding the recommendations.

However, there are some methodological parts that could still be improved, especially with the intention of being replicable. The abstract and the aim of the study still needs further clarification.

**********

what does this mean? ). If published, this will include your full peer review and any attached files.

If you choose “no”, your identity will remain anonymous but your review may still be made public.

**Do you want your identity to be public for this peer review?** For information about this choice, including consent withdrawal, please see our Privacy Policy

Reviewer #1: No

Reviewer #3: No

**********

[NOTE: If reviewer comments were submitted as an attachment file, they will be attached to this email and accessible via the submission site. Please log into your account, locate the manuscript record, and check for the action link "View Attachments". If this link does not appear, there are no attachment files.]

While revising your submission, please upload your figure files to the Preflight Analysis and Conversion Engine (PACE) digital diagnostic tool, https://pacev2.apexcovantage.com/ . PACE helps ensure that figures meet PLOS requirements. To use PACE, you must first register as a user. Registration is free. Then, login and navigate to the UPLOAD tab, where you will find detailed instructions on how to use the tool. If you encounter any issues or have any questions when using PACE, please email PLOS at figures@plos.org . Please note that Supporting Information files do not need this step.

# Author comment: Author response to Decision Letter 3

**DOI:** 10.1371/journal.pone.0335311.r007

19 Sep 2024

Review Comments to the Author

Please use the space provided to explain your answers to the questions above. You may also include additional comments for the author, including concerns about dual publication, research ethics, or publication ethics. (Please upload your review as an attachment if it exceeds 20,000 characters)

Reviewer #1: I went through the manuscript and the tracked changes document and the authors have responded adequately to my comments.

Reviewer #3: The article is now clearer; authors did a good work adding the recommendations.

However, there are some methodological parts that could still be improved, especially with the intention of being replicable. The abstract and the aim of the study still need further clarification.

Authors’ response:

We are thankful for the insightful review of this manuscript. For more clarification, explanations about the method and the purpose of the study were added. The corrections are done with track changes.

In the abstract:

Introduction: Given the critical perspective of intersectionality and its potential to identify the causes of inequalities, it has been employed increasingly in studies related to health. Despite the rich theoretical evidence about intersectionality, there is a need to consider this approach empirically. This study aimed to apply the intersectionality in practice for health policy makers and researchers seeking health inequalities. In this regard, we described the development of an intersectionality-based and context-specific intervention focusing on HIV testing uptake among Afghan immigrants in Iran.

Methods: This is an intervention development study. The intersectionality was used to design a peer-led intervention guided by the 2008 MRC framework. We undertook the following activities related to the three stages of the MRC framework: 1. Identifying the existing evidence (conducting a scoping review to investigate the application of intersectionality in designing and implementing health interventions; designing the checklist of applying the intersectionality in health interventions and programs) ; 2. Identifying and developing a program theory (conducting a realist review to identify why, how, and under what conditions peer interventions can improve HIV testing uptake among immigrants); and 3. Modeling process and outcomes (adapting the contextual factors identified by conducting a qualitative study and the realist review; extracting considerations regarding intersectionality principles using the checklist of applying the intersectionality in health interventions and programs; determining context specific, intersectionality-based and evidence-based intervention components for each of the intervention pathways).

Results: According to considerations regarding checklist of application of intersectionality principles, the intervention at the different individual, organizational, and policy levels with multiple strategies should be designed to respond to needs/conditions affecting HIV testing uptake among immigrants. We determined the peer-led intervention features to improve the use of HIV testing services in Afghan immigrants following the intersectionality principles, target group needs, and contextual conditions aiming to modify power structures. Intervention strategies included HIV information provision, support, community-based services, and structural interventions.

Attachment Submitted filename: Response to reviewers 3.docx

# Peer review: Decision Letter 3

**DOI:** 10.1371/journal.pone.0335311.r008

**Authors:** Magdalena Szaflarski

10 Oct 2025

From theory to practice of designing for diversity: applying intersectionality to improve HIV testing uptake

PONE-D-23-10867R3

Dear Dr. Majdzadeh,

We’re pleased to inform you that your manuscript has been judged scientifically suitable for publication and will be formally accepted for publication once it meets all outstanding technical requirements.

Within one week, you’ll receive an e-mail detailing the required amendments. When these have been addressed, you’ll receive a formal acceptance letter and your manuscript will be scheduled for publication.

An invoice will be generated when your article is formally accepted. Please note, if your institution has a publishing partnership with PLOS and your article meets the relevant criteria, all or part of your publication costs will be covered. Please make sure your user information is up-to-date by logging into Editorial Manager at Editorial Manager®  and clicking the ‘Update My Information' link at the top of the page. For questions related to billing, please contact billing support .

If your institution or institutions have a press office, please notify them about your upcoming paper to help maximize its impact. If they’ll be preparing press materials, please inform our press team as soon as possible -- no later than 48 hours after receiving the formal acceptance. Your manuscript will remain under strict press embargo until 2 pm Eastern Time on the date of publication. For more information, please contact onepress@plos.org.

Kind regards,

Magdalena Szaflarski, PhD

Academic Editor

PLOS ONE

Additional Editor Comments (optional):

Minor edit in Abstract, Introduction: "seeking health inequalities" should be something like "seeking to reduce health inequalities"

Reviewers' comments:

Reviewer's Responses to Questions

**Comments to the Author**

Reviewer #1: All comments have been addressed

Reviewer #3: All comments have been addressed

**********

2. Is the manuscript technically sound, and do the data support the conclusions?

Reviewer #1: Yes

Reviewer #3: Yes

**********

3. Has the statistical analysis been performed appropriately and rigorously?

Reviewer #1: Yes

Reviewer #3: N/A

**********

4. Have the authors made all data underlying the findings in their manuscript fully available?

The PLOS Data policy

Reviewer #1: Yes

Reviewer #3: Yes

**********

5. Is the manuscript presented in an intelligible fashion and written in standard English?

Reviewer #1: Yes

Reviewer #3: Yes

**********

Reviewer #1: All the comments have been addressed. The methodology in this paper is well-grounded. It uses a critical review of existing literature, an appraisal of HIV testing uptake programming, as well as modeling of processes and outcomes. The methods are clear and coherent, the theoretical basis is well-informed, and the paper is highly contextualized to their setting.

Reviewer #3: Authors did a great job reinforcing the document and adding all the comments of reviewers. It is an original and interesting article.

**********

what does this mean? ). If published, this will include your full peer review and any attached files.

If you choose “no”, your identity will remain anonymous but your review may still be made public.

**Do you want your identity to be public for this peer review?** For information about this choice, including consent withdrawal, please see our Privacy Policy

Reviewer #1: No

Reviewer #3: **Yes: ** ALMA ANGELICA VILLA RUEDA

**********

# Editor report: Acceptance letter

**DOI:** 10.1371/journal.pone.0335311.r009

**Authors:** Magdalena Szaflarski

PONE-D-23-10867R3

PLOS ONE

Dear Dr. Majdzadeh,

I'm pleased to inform you that your manuscript has been deemed suitable for publication in PLOS ONE. Congratulations! Your manuscript is now being handed over to our production team.

At this stage, our production department will prepare your paper for publication. This includes ensuring the following:

* All references, tables, and figures are properly cited

* All relevant supporting information is included in the manuscript submission,

* There are no issues that prevent the paper from being properly typeset

You will receive further instructions from the production team, including instructions on how to review your proof when it is ready. Please keep in mind that we are working through a large volume of accepted articles, so please give us a few days to review your paper and let you know the next and final steps.

Lastly, if your institution or institutions have a press office, please let them know about your upcoming paper now to help maximize its impact. If they'll be preparing press materials, please inform our press team within the next 48 hours. Your manuscript will remain under strict press embargo until 2 pm Eastern Time on the date of publication. For more information, please contact onepress@plos.org.

You will receive an invoice from PLOS for your publication fee after your manuscript has reached the completed accept phase. If you receive an email requesting payment before acceptance or for any other service, this may be a phishing scheme. Learn how to identify phishing emails and protect your accounts at https://explore.plos.org/phishing.

If we can help with anything else, please email us at customercare@plos.org.

Thank you for submitting your work to PLOS ONE and supporting open access.

Kind regards,

PLOS ONE Editorial Office Staff

on behalf of

Dr. Magdalena Szaflarski

Academic Editor

PLOS ONE
